# Role of liposomal amphotericin B in intensive care unit: an expert opinion paper

**DOI:** 10.1186/s44158-025-00236-z

**Published:** 2025-04-29

**Authors:** Linda Bussini, Michele Bartoletti, Matteo Bassetti, Andrea Cortegiani, Gennaro De Pascale, Francesco Giuseppe De Rosa, Marco Falcone, Maddalena Giannella, Massimo Girardis, Paolo Grossi, Malgorzata Mikulska, Paolo Navalesi, Federico Pea, Maurizio Sanguinetti, Carlo Tascini, Bruno Viaggi, Pierluigi Viale

**Affiliations:** 1 Infectious Diseases Unit, Hospital Health Direction, IRCCS Humanitas Research Hospital, 20089 Rozzano, Milan Italy; 2 Department of Biomedical Sciences, Humanitas University, Pieve Emanuele, 20072 Milan, Italy; 3 Division of Infectious Diseases, IRCCS Ospedale Policlinico San Martino, Genoa, Italy; 4 Division of Infectious Diseases, Department of Health Sciences (DISSAL), University of Genoa, Genoa, Italy; 5 Department of Precision Medicine in Medical Surgical and Critical Care, University of Palermo, Palermo, Italy; 6 Department of Anesthesia, Intensive Care and Emergency Policlinico Paolo Giaccone, University of Palermo, Palermo, Italy; 7 Department of Emergency, Intensive Care Medicine and Anaesthesia, Fondazione Policlinico Universitario A. Gemelli IRCCS, Rome, Italy; 8 Department, of Medical Sciences, Infectious Diseases, University of Turin, Turin, Italy; 9 Infectious Disease Unit, AOU Pisana PO Cisanello, University of Pisa, Pisa, Italy; 10 Department of Medical and Surgical Sciences, Alma Mater Studiorum, University of Bologna, Bologna, Italy; 11 Infectious Disease Unit, IRCCS Azienda Ospedaliero Universitaria Di Bologna, Bologna, Italy; 12 Anesthesia and Intensive Care Medicine, Policlinico Di Modena, University of Modena and Reggio Emilia, Modena, Italy; 13 Infectious and Tropical Diseases Unit, Department of Medicine and Surgery, University of Insubria - ASST-Sette Laghi, Varese, Italy; 14 Institute of Anesthesia and Intensive Care, University of Padua, Padua, Italy; 15 Clinical Pharmacology Unit, IRCCS Azienda Ospedaliero Universitaria Di Bologna, Bologna, Italy; 16 Department of Basic Biotechnological Sciences, Intensive and Perioperative Clinics, Catholic University of the Sacred Heart, Rome, Italy; 17 Infectious Diseases Clinic, Azienda Sanitaria Universitaria del Friuli Centrale (ASUFC), Udine, Italy; 18 ICU Department, Careggi Hospital, Florence, Italy

**Keywords:** Invasive fungal infections, Intensive care unit, Antifungal therapy, Liposomal amphotericin B, Critically ill patient, Aspergillosis, Candidiasis, Mold infections

## Abstract

**Introduction:**

Invasive fungal infections (IFI) are frequent in patients admitted to the intensive care unit (ICU). The use of first-line antifungals like triazoles or echinocandins may be limited by the global spread of multi-drug resistance species, drug–drug interactions, low organ penetration, and some safety concerns in case of multi-organ failure. Liposomal amphotericin B (L-AmB) is a polyene drug with a broad activity against mold and yeast and an acceptable safety profile. To outline the role of L-AmB in the treatment of IFI in critically ill patients, a panel of experts was invited to draw up an expert opinion paper on the appropriate place in therapy of L-AmB in different clinical scenarios of patients admitted to ICU.

**Methods:**

A multidisciplinary group of 16 specialists in infectious disease, microbiology, pharmacology, and intensive care elaborated an expert opinion document through a multi-step approach: (1) the scientific panel defined the items and wrote the statements on the management of IFI in ICU, (2) a survey was submitted to an external panel to express agreement or disagreement on the statements, and (3) the panel reviewed the survey and implemented the final document.

**Results:**

The final document included 35 statements that focused on epidemiology and microbiological rationale of the use of systemic L-AmB in critically ill patients and its potential role in specific clinical scenarios in the ICU.

**Conclusion:**

Systemic L-AmB may represent an appropriate therapeutic choice for IFI in ICU patients with different underlying conditions, especially when the use of first-line agents is undermined. This expert opinion paper may provide a useful guide for clinicians.

## Introduction

Invasive fungal infections (IFI) represent a life-threatening condition in patients admitted to the intensive care unit (ICU). The incidence of IFI in critically ill patients is rising, while attributable morbidity and mortality remain high [1, 2]. Reasons may lie in the higher complexity of care of patients with a major risk for IFI, including immunocompromised patients or those with severe medical or surgical comorbidities [3]. Other risk factors may be related to the extensive use of broad-spectrum antibiotics and invasive procedures which may favor tissue invasion by disrupting the integrity of epidermal and mucosal barriers [3, 4].

The epidemiology of IFI in the ICU is changing. Candidiasis is still the most common fungal infection, though a shift towards non-albicans species has been observed [5–7]. Moreover, the rate of invasive aspergillosis (IA) in critically ill patients is increasing [8]. Notably, the evidence of association between severe viral respiratory infections and IA warned about the emergence of new categories susceptible to IA without the “classical” risk factors like neutropenia or transplantation [9, 10]. Infections caused by rare molds like *Mucorales species*, *Fusarium species*, *Scedosporium species*, or *Lomentospora prolificans* are also standing out [11, 12]. In these cases, therapeutic management is challenging due to the lack of rapid diagnostic assays, the limited availability of antifungal susceptibility testing (AST), and poor clinical evidence about the effectiveness of current treatment options [13].

Finally, the worldwide spread of antifungal resistance to first-line agents like fluconazole, triazoles, and echinocandins is of great concern since currently alternative options are limited [14]. Outbreaks of azole-resistant *Candida albicans or C. parapsilosis* as well as echinocandin-resistant C. *parapsilosis* or *Pichia kudriavzevii (*formerly *C. krusei) in* ICUs are described worldwide [6, 14]. The recent emergence of nosocomial infections by *C. auris* is of great concern because of its environmental adaptability and multi-drug-resistant profile [15, 16]. Not least, the prevalence of azole-resistant *A. fumigatus* is increasing around the world also involving ICU patients with IA [17]. Even if many new antifungals are in the pipeline, robust data on their efficacy in critically ill patients are currently limited [18].

Liposomal amphotericin B (AmBisome®, L-AmB) is a polyene agent comprised of conventional amphotericin B included in liposomal unilamellar vesicles. By binding to ergosterol, amphotericin creates pores in the fungal cell membrane, leading to ion leakage and cell killing [19]. L-AmB has a wide spectrum of activity on numerous fungal species including *Candida species*, *Aspergillus species*, *Cryptococcus*, *Rhizopus species*, and other rare molds [19, 20]. Indeed, systemic L-AmB demonstrated a safer profile compared to conventional amphotericin B formulations, with a lower rate of nephrotoxicity and infusion reactions [21].

Guidelines consider L-AmB a reasonable alternative in case of refractory or resistant candidiasis and aspergillosis, as well as a first choice for mucormycosis and infections by other filamentous fungi [13, 22, 23]. However, the contemporary landscape of invasive mycosis in ICU is revealing tangible limitations in the use of current first-line agents [24].

For these reasons, a committee of specialists skilled in infections in critical care was called to elaborate an expert opinion document to address the use of systemic L-AmB for the most common IFI affecting patients admitted to ICU, focusing on specific clinical settings.

## Materials and methods

The scientific panel included 16 specialists in infectious diseases, microbiology, pharmacology, and intensive care selected based on their clinical expertise and scientific publications:

- Infectious diseases: P. Viale (scientific coordinator), M. Bartoletti (scientific secretary), M. Giannella (scientific secretary), M. Bassetti, F.G. De Rosa, M. Falcone, P. Grossi, M. Mikulska, and C. Tascini
- Intensive care: A. Cortegiani, G. De Pascale, M. Girardis, P. Navalesi, and B. Viaggi
- Clinical pharmacology: F. Pea
- Microbiology: M. Sanguinetti

The methodology for statement elaboration and approval was established in October 2023. A multi-step strategy was chosen to formulate an expert opinion document.

During the first meeting, the panel identified the clinical items and the open issues concerning the management of ICU patients at risk of invasive candidiasis and mold infections and the potential role of systemic L-AmB in these settings (Table 1).

**Table 1:** Initial items on the management of IFI in ICU and the role of systemic LAMB discussed during the first project meeting

Items	Clinical setting	
**Treatment strategies**	**Invasive candidiasis in ICU**	**Invasive mold in ICU**
	- Use of empirical therapy, particularly in abdominal candidiasis - Use of empirical therapy based on clinical criteria - Use of pre-emptive strategy based on colonization - Use of pre-emptive strategy based on biomarkers - Early withdrawal of antifungal treatment	- Use of empirical therapy - Use of empirical therapy based on clinical criteria
**Microbiological considerations**	- Role of biomarkers for non-albicans species	- Role of biomarkers in early diagnosis - Role of PCR in early diagnosis - Universal versus targeted use of microbiological diagnostics
**Pharmacological considerations on systemic LAMB**	- Dosage - Safety - Comparison with other antifungal drugs and drug-drug interactions	
**Patient setting**	- SARS-CoV-2 or influenza virus infections - patients on therapy with corticosteroids or immunomodulatory drugs - Chronic obstructive pulmonary disease - Diabetes - End-stage liver disease - Solid organ transplantation - Hematologic malignancy - Abdominal surgery	

Then, the panel members were divided into subgroups based on specific expertise to produce one or more statements for each item or patient setting (Table 1). The formulation of each statement was supported by a narrative review.

The initial statements were finally reviewed by the whole panel until a general agreement was reached.

In the second step, the statements were tested by an external panel of Italian physicians selected based on proven clinical experience and scientific relevance in the field of infections in the ICU. Of 67 clinicians invited, 51 participated in an online survey. The external panel expressed the level of agreement or disagreement with each statement through a 9-point scale, where 0 points corresponded to “strongly disagree” and 9 points to “strongly agree”.

The results of the survey did not aim to change the content of the statements; however, the statements receiving less than 8.0 of the average rate of agreement were discussed by the scientific panel before their inclusion in the final document.

## Results

The scientific panel formulated 35 statements on the general management of IFI in the ICU and the role of LAMB. Table 2 details the statements on the use of systemic LAMB. Overall, the statements received a high level of agreement (median rate 8.0) from the external panel. The statements receiving an average score < 8.0 are marked with an asterisk (*) in the text. These statements and their revisions are shown in Table 3.

**Table 2:** Summary of statements on the use of systemic LAMB in ICU

**Pharmacology**	The recommended dose of L-AmB for most indications in critically ill septic patients is 3 mg/kg, with a maximum of 5 mg/kg/day (a dose ceiling of 500 mg is recommended in patients weighing > 100 kg). Daily doses of L-AmB > 5 mg/kg are not associated with a significant benefit in terms of clinical outcome in any type of fungal infection and could increase the risk of nephrotoxicity and hypokalemia. However, a single 10 mg/kg dose could be considered for treating visceral leishmaniasis and/or cryptococcal meningitis
	The risk of nephrotoxicity of L-AmB at a dose of 3–5 mg/kg/day is much lower than that of amphotericin B deoxycholate
	In critically ill patients with renal dysfunction and/or requiring hemodialysis or continuous renal replacement, no dosing adjustment of L-AmB is necessary due to the fact that its elimination is non-renal and the incidence of adverse events did not markedly differ from non-RRT groups
**Therapeutic approach to mold infections in patients with severe viral pneumonia, chronic corticosteroids or immunomodulatory therapy, COPD, diabetes, and end-stage liver disease**	Anti-mold therapy with L-AmB could be preferable over azoles in case of treatment failure and could be proposed as the first-line option (i) in geographic areas with a high prevalence of azole resistance (ii) in patients at higher risk for hepatotoxicity (i.e., end-stage liver disease) in subjects taking drugs having clinically relevant drug-drug interactions vs. azoles (iv) in setting having no possibility of performing voriconazole TDM
	The interindividual pharmacokinetic variability of L-AmB in critically ill patients is expected to be limited so that TDM is not needed
	L-AmB demonstrated efficacy in the treatment of mucormycosis with various organ involvement patterns. The daily dose should be 5 mg/kg per day
**SOT**	In SOT recipients, a targeted (risk-based) approach to antifungal prophylaxis is recommended. Clinically relevant drug-drug interactions, safety concerns, and rates of breakthrough infections are all issues to be taken into account when choosing an antifungal agent for prophylaxis. In this regard, L-AmB may be considered a suitable option
	Drug-drug interactions with immunosuppressive drugs could sometimes represent a relevant issue when treating IFI with azole antifungals after SOT. In this regard, L-AmB could be a valuable alternative option for the empirical treatment of IFI
	Regarding IC in SOT recipients, L-AmB could be considered a reasonable alternative to echinocandins
**Hematologic malignancy**	Patients with hematologic malignancies receiving mold-active azole prophylaxis who develop suspected or documented breakthrough IFI should receive treatment with L-AmB and promptly undergo a complete diagnostic work-up
	Patients with hematologic malignancies admitted to the ICU and having IFI with no possibility for TDM for azoles and/or at high risk of azole-related drug–drug interactions should receive treatment with L-AmB
	Considering the high risk of IFI and wide spectrum of fungal pathogens in certain hematology patients (with prolonged neutropenia or after allogeneic HSCT), empirical therapy with L-AmB can be useful in patients admitted in ICU with clinical suspicion of IFIs while completing diagnostic work-up and it should be discontinued if the suspicion of IFI is not confirmed
**Abdominal surgery**	In patients with IAC, the choice of empirical antifungal therapy should be guided by host, microbiological ad epidemiological variables. L-AmB could be considered first-line therapy in cases of intra-abdominal infection with sepsis/septic shock, the risk for *N. glabratus* and *C. parapsilosis* infections, or previous therapy with echinocandins
	Echinocandins could be used as a first-choice treatment in non critically ill patients. However, recent pharmacokinetic/pharmacodynamic evidence suggested that exposure to the ascitic fluid may be suboptimal and may cause breakthrough resistance, especially in the case of non-albicans etiology
	Combination therapy with L-AmB and an echinocandin should be considered a rescue therapy in the case of *C. auris* etiology

**Table 3:** Review of statements receiving an average rating < 8.0 in the external panel survey

Section	Statement N°	Average rating	Main critical points by an external panel	Review by the scientific committee
Microbiology	8	7.8	• Low diagnostic yield of culture	• Despite diagnostic limits, cultures remain the gold standard for species identification and for susceptibility tests
	10	7.7	• Lack of standardization of molecular tests • Unavailability of susceptibility tests and molecular techniques in many laboratories	• The SP agreed on the lack of standardization of molecular diagnostics. Moreover, the high sensitivity may bring false positive results and consequently over-treatment • The SP agreed on the current unavailability of molecular techniques in most national centers. However, this debate should trigger the efforts to implement lab resources and work on the standardization of methodic
Molds and SARS-CoV-2 and/or Influenza virus coinfections	14	7.2	• Clarify the use of L-AmB as pre-emptive or empirical therapy in this setting	• The SP endorsed the use of L-AmB as empirical therapy in patients with clinical and microbiological criteria. Only in patients with additional risk factors for mold infection, the use of L-AmB as pre-emptive treatment may be considered
	15	7.9	• Further studies with adequate design are needed to evaluate the efficacy and safety of any kind of prophylaxis • The role of L-AmB by aerosol as anti-mold prophylaxis or treatment is controversial	• The use of antifungal prophylaxis in patients with viral pneumonia is not justified by current clinical data • The SP agreed that the efficacy and safety of L-AmB aerosol for the prophylaxis or treatment should be confirmed by adequately sized studies yet
Diabetes	19	7.9	• Daily dose of L-AmB in mucormycosis may be extended up to 10 mg/kg/day	• The most recent evidence on mucormycosis observed that the use of high doses > 5 mg/kg/day did not improve survival [62]
End-stage liver disease	20	7.7	• ACLF and decompensated cirrhosis may have different weights as risk factors for IA	• End-stage liver disease is a well-known risk factor for IA [89]. Data on ACLF are more scarce; however, ACLF is emerging as a risk factor for IFI with a high mortality rate [90, 91]
Therapeutic approach to mold infections in patients with severe viral pneumonia, chronic corticosteroids or immunomodulatory therapy, COPD, diabetes, and end-stage liver disease	21	7.9	• Use of TDM may be justified only in patients receiving voriconazole, while it may be not needed for isavuconazole	• Recent data showed that ICU patients may have significantly lower isavuconazole blood levels compared to non-ICU patients, especially with BMI > 25 kg/m^2^, bilirubin > 1.2 mg/dL, and no hematology malignancy. Therefore, isavuconazole TDM may be used in an ICU setting [95]
SOT	23	7.4	• Use of L-AmB as prophylaxis in SOT in controversial	• The SP agreed that L-AmB is not registered for antifungal prophylaxis in SOT and there is no clear indication on the appropriate dosing. However, emerging data may sustain the use of LAMB for this purpose [114]
	24	7.9	• Drug-drug interaction between azoles and immunosuppressive agents may be properly managed with TDM	• L-AmB is considered the best option in patients in whom first-line therapy is associated with important drug–drug interaction
	25	7.7	• Use of L-AmB for IC in SOT is a preferable option in case of difficult-to-treat infection site (e.g. IAC) or *Candida spp* with lower susceptibility to echinocandins	• The SP agreed on the use of L-AmB as an alternative agent for IC in SOT
	26	7.9	• The content of the statement is unclear	• The SP agreed and reviewed the text of the statement
Hematology malignancy	30	7.8	• Clarify the use of L-AmB as pre-emptive or empirical therapy in this setting	• In high-risk hematologic patients admitted to ICU, empirical therapy with L-AmB can be in case of clinical suspicion of IFI while ongoing diagnostic work-up and discontinued if the suspicion is not confirmed
Abdominal surgery	32	7.9	• L-AmB could be considered a first-line therapy in cases of septic shock, not sepsis	• The SP considered L-AmB a front-line option in patients with both sepsis and septic shock [142]
	33	7.6	• Evidence on the correlation between antifungal PK/PD issues and clinical outcomes is scarce	• The SP agreed that evidence on the correlation between PK/PD parameters and clinical outcome is limited and further clinical trials are needed. However, the SP considered concerning recent pharmacological data that documented an association between suboptimal exposure to echinocandins in ascitic fluid and emerging breakthrough resistance [149]
	35	7.6	• Data supporting BDG-driven stopping strategies are too few	• The SP considered BDG-driven strategies suitable to the most of manifestations of IC. However, in some conditions like deep-seated intrabdominal infections or *C. parapsilosis* infections, BDG may have lower sensitivity; thus, BDG-driven strategies may not be a reliable approach

*ACLF* Acute-on-Chronic Liver Failure, *BDG* Beta-D-Glucan, *BMI* body mass index, *COPD* chronic obstructive pulmonary disease, *GM* Galactomannan, *IAC* intra-abdominal candidiasis, *IC* Invasive Candidiasis, *ICU* Intensive Care Unit, *IFI* Invasive Fungal Infection, *L-AmB* liposomal amphotericin B, *PK/PD* pharmacokinetics/pharmacodynamics, *SP* Scientific Panel, *SOT* solid organ transplantation, *TDM* therapeutic drug monitoring

### General statements about the role of liposomal amphotericin B

1. Considering the scientific evidence currently available, making a univocal decision about treatment choice for every IFI is basically impossible. Moreover, patients admitted to ICU may have specific risk factors for IFI as well as severe impairment of one or more organ functions may affect the antifungal treatment. Thus, every decision regarding antifungal drug choice for severe or complicated infections in critically ill patients should be individualized based on the simultaneous evaluation of epidemiological, microbiological, pharmacological, and clinical variables.
2. Inside the antifungal armamentarium, L-AmB represents a valuable choice in several different settings and fungal infections, thanks to its wide antifungal spectrum of activity, limited propensity to develop resistance, low impact in terms of drug-drug interactions, good capability of overcoming biological barriers, no need for therapeutic drug monitoring (TDM) and acceptable safety profile.

### Microbiology

#### Background

The role of microbiological biomarkers in diagnosing IFI in the ICU is highly debated. The characteristics, strengths, and limits of the main tests are shown in Table 4.While molecular and antigen-based methods have improved the speed and sensitivity of diagnosing IFI, the classical culture of clinical samples remains important for confirming the diagnosis and for species identification [39]. However, classical cultural techniques may take several days to yield results, which can delay the initiation of target therapy. Moreover, the sensitivity of cultures may be influenced by various factors, including previous exposure to antifungals or accuracy in sample collection [40] (Table 4).Standardized antifungal susceptibility testing (AST) includes different methods performed on positive cultures; however, new resistance molecular tests are getting into clinical practice [25]. Although not ubiquitously available, the use of AST may be critical to guide antifungal therapy in the ICU. Patients admitted to ICU may have an increased risk for resistant infections, due to both individual factors (i.e., immunosuppression, previous antifungal exposure, or long hospitalization) [26, 27] and environmental conditions (i.e., large use of azoles in agriculture, the crisis of ICUs during COVID19 pandemic) [14, 17]. Of note, outbreaks of fluconazole-resistant *C. parapsilosis*, multi-drug-resistant *C. auris*, or azole-resistant *A. fumigatus* have been reported in ICUs worldwide [17, 26, 28].

**Table 4:** Characteristics of the main microbiological tests for IFI in ICU

**General characteristics**		**Culture**	**Polymerase Chain Reaction**	**Galactomannan antigen**	**Beta-D-glucan**	**Other biomarkers (mention)**
		•Gold standard for diagnosis of invasive fungal infection •Performed on various samples (blood, sputum, BALF, CSF…) with different level of sensitivity • Allow the execution of susceptibility test	• Direct test targeting specific DNA sequences of various fungal species • Performed on various samples (blood, sputum, BALF, CSF…) with high sensitivity • Allow testing main molecular mechanisms of antifungal resistance.	• Indirect test detecting polysaccharide antigen of Aspergillus cell wall released into the blood and other body fluids even in the early stages of fungal invasion • Generally performed on BALF and serum	• Indirect test detecting (1→3)-β-D-glucan, a component of the cell walls of various fungi, particularly *Candida species*, early released into the bloodstream when fungal cells break down or during active fungal growth. • Generally performed on serum, but also possible on other materials (i.e. BALF, peritoneal fluid)	(see clinical application)
**Invasive candidiasis**	**Clinical application in ICU**	• Diagnosis confirmation [29, 30] • By providing identification of species and susceptibility profile, it is essential for choosing the targeted antifungal therapy and dosage [29, 31] • Negative follow up blood culture during treatment of candidemia underline positive response to treatment and may define duration of therapy [32] • Follow-up blood cultures after discontinuation of therapy could reveal any potential relapse of candidemia, especially in high-risk patients [29]	Not commonly used	Not used	• If candidemia is suspected, serum BDG may provide earlier diagnosis than traditional culture methods [33] • Its high NPV allow early interruption of an empirical antifungal therapy in case of negative result [34] • Serial dosing of BDG level may appraise of the efficacy of antifungal therapy and guide treatment duration [35] • higher BDG value at diagnosis is associated with a higher risk of therapeutic failure [35]	•.Mannan Antigen (Mn): cell wall component of Candida spp, Combination of Mn and anti-mannan antibody (Mn/A-Mn) testing demonstrated high sensitivity and specificity in IC diagnosis [36] • Candida albicans germ tube antibody (CAGTA): detection og IgG antibodies against several superficial antigens of the germ tubes of C. albicans by indirect immunofluorescence. Higher performance when used in combination with other biomarkers of IC (i.e BDG) [37, 38]
	**Limits**	• High turn-around time (48–72 h up to 8 days) may affect early diagnosis and treatment [29, 39] • sensitivity may be influenced by various factors (e.g. prior therapy, volume of sample cultured, timing of sample collection from the onset of symptoms) [29, 39]	-	-	• Not suitable for early identification of patients at risk of IC due to possible false positive results, (i.e. in presence of heavy mucosal or skin fungal colonization, albumin and immunoglobulin administrations, haemodialysis, packing with surgical gauzes, non-glucan-free laboratory equipment [40] • Possible false negative results with some *Candida species* (e.g *N. glabratus*) [40] • Lower sensitivity in candidiasis than candidemia [29] • BDG guided antifungal treatment did not demonstrated to improve survival among ICU sepsis patients with risk factors for IC [41]	
**Invasive pulmonary aspergillosis**	**Clinical application in ICU**	• Diagnosis of proven infection on biopsy (culture + microscopy)[39, 42] • Diagnosis of possible infection on BALF/tracheal aspirate [42] • By providing identification of species and susceptibility profile, it is essential for choosing the targeted antifungal therapy and dosage [43] • Monitoring treatment response by evidencing changes in the growth pattern or susceptibility profile [44]	• Diagnosis of proven infection on biopsy (PCR +microscopy) [45, 46] • PCR on BALF may be a complementary tool for early diagnosis of IPA [42, 45] • Rapid identification of triazole resistance [47]	• Diagnosis of possible infection on BALF or serum [43] • High sensitivity on BALF allows early diagnosis in patients with consistent clinical and radiological signs [42, 43, 48, 49] • Serial quantitative GM levels in BALF may be valuable for assessing the response to therapy [43, 50, 51]	• Both BALF and serum BDG may contribute to the microbiological diagnosis of pulmonary aspergillosis [39]	• Aspergillus-specific lateral-flow device (LFD): rapid diagnostic test based on monoclonal antibody able to detect an extracellular mannoprotein antigen of Aspergillus species. May be performed on serum or BALF [52]
	**Limits**	• Slow turn-around time, up to several days [53] • Susceptibility test is generally available in few Centers [53]	• False-positive results in case of colonizing or non-vital organisms [45, 54]	• Possible false positive results with some medications (e.g., piperacillin-tazobactam) and other fungal infections (e.g. talaromycosis); the result needs carefully interpretation along with clinical and radiological features [55] • Controversial association between GM level and response to therapy [51] • Quantitative serum GM testing demonstrated reduced sensitivity compared with GM in BALF [50]	• Possible false positive results [53]	
**Other fungal infections**	**Clinical application in ICU**	• Diagnosis confirmation in case of mold infections, species identification, and possibly susceptibility test [13]	• Identification of rare fungal species, especially slow-growth fungi [13, 56] • Earlier diagnosis than traditional culture methods [56] • Useful in case of deep-seated infections (CNS Infections) [56, 57]	Not conventionally used	• Serum BDG is commonly used for diagnosis of *Pneumocystis jirovecii* pneumonia in immunocompromised patients [58] • Negative result of serum BDG excludes diagnosis of PJP [58]	• Cryptococcal polysaccharide antigen on serum or CSF or Histoplasma antigen on urine demonstrated good sensitivity and specificity in diagnosis of disseminated disease in immunocompromised hosts [59–61]
	**Limits**	• Low diagnostic yield, especially on blood [13] • High turn-around time[13] • Susceptibility test is available in few Centers[13]	• Possible false positive results (e.g. colonization) [62] • Possible false negative results [13] • Available in few Centers [62] • Lack of standardization of tests [62]		• Possible false positive results [58]	

*BALF* bronchoalveolar lavage fluid, *BDG* Beta-D-Glucan, *CNF* central nervous system, *CSF* cerebrospinal fluid, *GM* Galactomannan, *PJP* *Pneumocystis jirovecii* pneumonia, *PCR* Polymerase Chain Reaction

#### Statements

3. Epidemiology of IFI is changing due to several factors including the better performance of microbiological diagnosis, the increased numbers and diversity of susceptible patients (i.e., COVID-19, biologics), the exposure to antifungals both in the individual and in the environment, and the changing climate. Emerging infections/resistance patterns mandate the need for timely and accurate diagnostics as well as for species identification and detection of antifungal resistance. In other terms, access to mycology laboratory expertise is key for the proper management of IFI.
4. Despite the considerable variability in populations and reference criteria employed, investigations into laboratory assays for diagnosing IPA consistently revealed GM from BALF a superior diagnostic accuracy over serum GM. Additionally, both BALF and serum BDG demonstrated less-than-ideal specificity.
5. Quantitative GM testing, especially in BALF, is a valuable and widely used biomarker for the diagnosis of IA in the ICU. However, results should always be interpreted in the context of clinical, radiological, and other laboratory findings.
6. Polymerase chain reaction test from BALF can be a valuable tool for the diagnosis of aspergillosis in the ICU, especially in high-risk and immunocompromised patients. However, the sensitivity and specificity of PCR can vary depending on the patient population and the specific PCR method used. Standardization of protocols for DNA extraction and PCR assays is important for improving diagnostic accuracy (i.e., species identification, resistance genotypes…).
7. Beta-glucan testing can be a valuable tool for diagnosing IC with or without candidemia in ICU patients. Based on its high NPV, BDG should be included in the decision tree aimed to exclude systemic *Candida* infection.
8. Classical culture plays an important role in the diagnosis of IA by providing a definitive identification of the pathogen and guiding appropriate treatment strategies.*
9. Blood cultures are the gold standard for diagnosing candidemia in the ICU. They are mandatory not only for microbiological diagnosis but also for the identification of causative species, testing sensitivity, and monitoring the timing of treatment response.
10. Polymerase chain reaction tests, including pan-fungal PCR assays and plasma cell-free DNA fungal PCR panels, can provide sensitive and specific detection of various fungal pathogens beyond Candida and Aspergillus species. These tests have the potential to aid in the early and accurate diagnosis of fungal infections, leading to improved patient outcomes. *

### Pharmacology

#### Background

Liposomal amphotericin B is characterized by a concentration-dependent fungicidal activity [20]. In experimental animal models, the main determinant of efficacy was found to be the maximum concentration (Cmax)/minimum inhibitory concentration (MIC) ratio [19]. Studies evaluating the pharmacokinetic profile of L-AmB in critically ill patients are rather limited [19, 63, 64]. From the available data, there is a certain interindividual variability, but this does not appear to be attributable to any specific pathophysiological condition. No correlation was found with renal function, albuminemia, and/or Sequential Organ Failure Assessment (SOFA) score [63]. The Cmax and the area under curve (AUC) levels achievable in critically ill patients during treatment with doses of L-AmB of 3–5 mg/kg/day are quite like those found in healthy volunteers and/or other patient populations [63]. Furthermore, maximum concentration (Cmax) and AUC do not appear to be influenced by the application of continuous renal replacement therapy (CRRT) [64, 65]. It has been reported some case reports that during Extra-Corporeal Membrane Oxygenation (ECMO) a certain increase in the volume of distribution (Vd) can occur [66–68]; however, available PK data on LAmB in ECMO are few and controversial [69]. Some authors suggested that using doses per/kg of total body weight in patients with morbid obesity could cause an increased risk of nephrotoxicity, especially at a dose of 5 mg/kg/day [70–72]. Overall, considering these data, it is believed that in critically ill patients, the maximum dose of L-AmB could be 5 mg/kg/day with a ceiling dose of 500 mg in patients weighing > 100 kg.

A recent meta-analysis analyzed 10 single- or double-blind, randomized, controlled, clinical trials that included a total of 1661 patients treated with high doses of L-AmB (> 5 mg/kg/day; range 6–15 mg/kg/day) compared to standard doses of L-AmB (3 mg/kg/day, 4 studies) or of amphotericin B deoxycholate (0.7–1 mg/kg/day, 3 studies) or of posaconazole (200 mg q6h oral suspension, 1 study) or in the absence of antifungals (1 study) [73]. Therapeutic efficacy was evaluated as the primary outcome, while mortality, survival ≥ 10 weeks, and adverse reactions were evaluated as secondary outcomes. The use of doses of L-AmB > 5 mg/kg/day was not associated with an advantage in terms of clinical outcome. In particular, the analysis of the 3 comparative studies concerning the treatment of IA did not demonstrate any statistically significant advantage for the high doses in terms of therapeutic efficacy (OR = 0.35, 95% CI 0.06–2.12, *P* = 0.25). By contrast, the use of high doses was associated with an increase in mortality, a reduction in long-term survival (≥ 10 weeks, OR = 0.57, CI 95% 0.34–0.94, *P* = 0.03) and an increase in adverse events (including renal failure). Out of specific indications for using a high-dose single shot or loading dose of L-AmB [74], the only setting in which the hypothesis of using high daily doses of L-AmB > 5 mg/kg continues to be postulated is that of mucormycosis [75]. However, a recent retrospective, multicenter study analyzing 82 confirmed and probable cases of mucormycosis collected between 2015 and 2022 in 51 Japanese hospitals concluded that the use of high doses > 5 mg/kg/day did not improve survival. Conversely, a single 10 mg/kg dose may be considered a good option for treating visceral leishmaniasis [76, 77] and/or cryptococcal meningitis [74].

The risk of nephrotoxicity of amphotericin B deoxycholate is due to the accumulation that occurs in the renal tubular cells with this formulation. By contrast, L-AmB has a much lower Vd than the deoxycholate formulation, and this results in a lower propensity of accumulation and a lower risk of toxicity. This is because the liposome, by acting as a reservoir and by remaining intact until contact with the fungal membrane, retains the amphotericin B in its wall and may prevent its accumulation at the renal level [19]. In a comparative meta-analysis against amphotericin B deoxycholate including 10 studies with a total of 2172 participants, L-AmB was found to be significantly safer than conventional amphotericin B in terms of increase in serum creatinine over twofold the baseline value (RR 0.49, 95% CI from 0.40 to 0.59) [21]. In the specific context of critically ill patients, a recent prospective phase 2 study enrolling 40 adult patients at high risk of intra-abdominal candidiasis (IAC) after major abdominal surgery demonstrated that pre-emptive therapy with a single 5 mg/kg dose of L-AmB, followed by prompt withdrawal in case of negative baseline BDG result, was a safe and effective approach [78]. A retrospective clinical study evaluated the usage and occurrence of adverse reactions during L-AMB therapy in patients undergoing renal replacement therapy (RRT). In total, 24, 19, and 842 cases were included in the hemodialysis (HD), CRRT, and non-RRT groups, respectively. After propensity score matching, the average daily and cumulative dose, treatment duration, and dosing interval for L-AMB were not significantly different and the incidence of adverse events did not markedly differ among the groups [79].

#### Statements

11. The recommended dose of L-AmB for most indications in critically ill septic patients is 3 mg/kg, with a maximum of 5 mg/kg/day (a dose ceiling of 500 mg is recommended in patients weighing > 100 kg). Daily doses of L-AmB > 5 mg/kg are not associated with a significant benefit in terms of clinical outcome in any type of fungal infection and could increase the risk of nephrotoxicity and hypokalemia. However, a single 10 mg/kg dose could be considered for treating visceral leishmaniasis and/or cryptococcal meningitis.
12. The risk of nephrotoxicity of L-AmB at a dose of 3–5 mg/kg/day is much lower than that of amphotericin B deoxycholate.
13. In critically ill patients with renal dysfunction and/or requiring hemodialysis or continuous renal replacement, no dosing adjustment of L-AmB is necessary due to the fact that its elimination is non-renal and the incidence of adverse events did not markedly differ from non-RRT groups.

### Specific clinical settings

#### Molds and SARS-CoV-2 and/or influenza virus coinfections

### Background

Both severe influenza and severe/critical COVID-19 are associated with a higher risk for invasive pulmonary aspergillosis (IPA). These conditions were named influenza-associated pulmonary aspergillosis (IAPA), and COVID-19-associated pulmonary aspergillosis (CAPA), respectively. Complex pathophysiological interactions involving viruses, the damaged lung parenchyma, immune cells, and *Aspergillus *spp. were demonstrated. The virus-induced injury and the following activation of the immune cells can facilitate the progression from contamination with Aspergillus conidia to tissue invasion and potentially lead to the angio-invasive phase [80, 81]. The ability of the macrophages to destroy Aspergillus conidia seems to be impaired in case of high viral burden [80, 81]. For this reason, IPA associated with respiratory virus is considered a specific entity in critically ill patients, called virus-associated pulmonary aspergillosis (VAPA) [10, 82]. Clinical practice guidelines and guidance documents for the diagnosis and management of both IAPA and CAPA were released [83, 84]. In patients with severe viral pneumonia, respiratory failure, and need for respiratory support, a diagnosis of IPA should be pursued. Galactomannan optical density index (ODI) on BALF or other deep respiratory specimens should be measured in every patient at ICU admission and serially once a week. As for the use of antifungal prophylaxis in this setting, current clinical evidence does not justify this practice [85, 86]; indeed, the incidence of CAPA and IAPA may vary significantly across different geographical areas [87].

### Statements

14. In patients with severe viral pneumonia, respiratory failure, need for respiratory support and no other risk factors for IPA, initiation of anti-mold treatment should be postponed until microbiological diagnostic criteria have been addressed. On the contrary, in patients with severe viral pneumonia and other risk factors for IPA (e.g., corticosteroid therapy, COPD, immunosuppression) empiric treatment should be considered. *
15. Widespread anti-mold prophylaxis in critically ill patients with viral pneumonia is not currently justifiable by available evidence. *

#### Patients on therapy with corticosteroids or immunomodulatory drugs

### Background

The chronic use of high-dose corticosteroids has been defined as a risk factor for pulmonary aspergillosis for decades. Indeed, chronic therapy with steroids is one of the host criteria of the EORTC-MSG and AspICU algorithm for the diagnosis of IPA [88]. More recently, corticosteroid therapy was found as a peculiar risk factor for developing IAPA in patients with severe influenza [9]. Although dexamethasone was demonstrated to reduce mortality in severe/critical COVID-19 patients, its use was associated with a higher risk of developing CAPA in several observational studies [89]. Dexamethasone seems to reduce the macrophages’ ability to prevent *A. fumigatus* germination, which may be correlated with fast fungal growth, destruction of macrophages, and induction of an anti-inflammatory cytokine profile. Moreover, other drugs associated with reduced mortality in severe/critical COVID-19 patients, such as anti-interleukin (IL)−6 (e.g., tocilizumab) were associated with a higher risk of developing CAPA [90].

### Statement

16. Chronic therapy with corticosteroids or immunomodulatory drugs should lead to a high index of suspicion of IPA in critically ill patients with pulmonary infiltrates, driving an early diagnostic approach.

#### Chronic obstructive pulmonary disease

### Background

Patients with COPD are recognized as at higher risk of developing IPA. However, in critically ill patients with COPD, respiratory failure, lung consolidations, and positive *Aspergillus* tests from the respiratory tract (either culture or GM), the discrimination between *Aspergillus* colonization or infection may be hard. Since IPA prognosis in critically ill patients is quite poor, the use of algorithms including all those findings may foster early diagnosis and appropriate antifungal therapy [88].

Furthermore, emerging evidence suggests that adopting a pre-emptive strategy in critically ill non-neutropenic patients, particularly those with COPD, may result in significant clinical benefit. This pre-emptive approach is based on the early use of microbiological biomarkers (e.g., GM in respiratory samples, *Aspergillus* PCR, and BDG assay) and consistent lung imaging [91]. More recently, a risk‑predictive model for IPA in patients with acute COPD exacerbation was proposed, which included serum albumin < 30 g/L, GOLD severity classes III–IV, steroid treatment in the previous three months, and broad-spectrum antibiotics for more than 10 days in the last month [92].

### Statement

17. Patients with COPD are at higher risk of developing IPA. Therefore, a prompt diagnostic approach must be pursued in any case of infection-related respiratory worsening.

#### Diabetes

### Background

Diabetes mellitus is the leading comorbidity in immunocompetent patients with mucormycosis [93]. Considering that the global prevalence (age-standardized) of diabetes rose from 4.7 to 8.5% in the last 50 years, an estimated 500 million adults are living with diabetes today, with the greatest increment in countries with a valuable circulation of Mucorales such as China, Brazil, Japan, Mexico, Egypt, and India [93–95]. Rhino-orbital-cerebral mucormycosis is the most frequent presentation among these patients, even in the absence of underlying conditions of immunosuppression [94]. Of note, COVID-19 pneumonia was described as an adjunctive risk factor for mucormycosis in diabetic patients [96]. The first step of management of mucormycosis should be a high clinical and radiological suspicion and prompt performance of both microbiological and histopathological investigations on tissue samples. However, the severity of infection along with the long processing time of diagnostic tests on tissue imposes an early introduction of empirical antifungal therapy [97]. Moreover, early antifungal administration seems not to affect the yield of histopathology or cultures [98]. The first-line agent for any organ involvement should be high-dose L-AmB and slow dose increment should be avoided [97, 99]. However, a recent retrospective study on 82 patients with mucormycosis did not show better survival of patients receiving L-AmB dose > 5 mg/kg/day versus 5 mg/kg/day [100]. The use of isavuconazole or posaconazole is mainly recommended as second-line or salvage therapy [97, 101]. Of note, clinical data on the efficacy of a combination therapy with amphotericin plus azoles or echinocandins are controversial to support this strategy [97, 102, 103]. Surgery is a cornerstone of the treatment and should be performed whenever feasible [104, 105]. Finally, correction of the predisposing factor including achievement of an adequate glycemic control is critical for the containment of the infection [106].

### Statements

18. In the last 50 years, diabetes has evolved as one of the major risk factors for mucormycosis, while more recently, underlying malignancy, severe immunodepression conditions, and SARS-CoV-2 infection emerged as important risk factors.
19. L-AmB demonstrated efficacy in the treatment of mucormycosis with various organ involvement patterns. The daily dose should be 5 mg/kg per day.*

#### End-stage liver disease

### Background

Increasing data are documenting cases of IPA among critically ill patients with acute liver failure or chronic cirrhosis [107]. Susceptibility to IPA may be related to immune dysfunction associated with liver failure, affecting both innate and adaptive immunity, along with the low platelet count, which has a growth-inhibiting effect on *Aspergillus species* [108]. The real incidence of IPA in patients with acute liver failure is probably underestimated, except for severe alcoholic hepatitis where incidence is about 15% and mortality almost 100% [109–111]. The rate of IPA in patients with end-stage liver disease achieved up to 14%, including those with Child–Pugh score C, a high model for end-stage liver disease (MELD) values/liver failure grade and concomitant COPD. Most of them require invasive mechanical ventilation and renal replacement therapy [109]. Interestingly, in a large cohort of cirrhotic patients admitted to the ICU (*n* = 986), 60 had a positive respiratory culture for *Aspergillus *spp, with a 28% rate of proven/putative IPA and 71% mortality rate [112]. Indeed, in critically ill patients with liver failure (especially Child C cirrhosis), the presence of compatible clinical signs and a positive GM antigen (ODI ≥ 1) on BALF, may support the diagnosis of probable IPA [88].

The ESCMID-ECMM-ERS guidelines recommended the use of L-AmB for IPA in patients with liver insufficiency [55]. This consideration relies on the possible hepatotoxicity of azole treatment in the presence of liver failure [80].

### Statement

20. In critically ill patients, acute on chronic liver failure and decompensated cirrhosis are recognized main risk factors for IA.*

#### Therapeutic approach to mold infections in patients with severe viral pneumonia, chronic corticosteroids or immunomodulatory therapy, COPD, diabetes, and end-stage liver disease

### Statements

21. Anti-mold therapy with L-AmB could be preferable over azoles in case of treatment failure and could be proposed as the first-line option (i) in geographic areas with a high prevalence of azole resistance (ii) in patients at higher risk for hepatotoxicity (i.e., end-stage liver disease) in subjects taking drugs having clinically relevant drug-drug interactions vs. azoles, (iii) in setting having no possibility of performing voriconazole TDM.*
22. The interindividual pharmacokinetic variability of L-AmB in critically ill patients is expected to be limited so that TDM is not needed.

#### Solid organ transplantation

### Background

The incidence of IFI and distribution of pathogens vary according to the type of transplant and local epidemiology [113, 114]. IFI incidence is usually higher after small bowel, lung, and liver transplantation compared with other types of SOT [113–115]. *Candida species* and *Aspergillus species* are the main pathogens. Overall IC is the prevalent type of IFI after abdominal transplantation, while IA is the main IFI after lung transplantation [113–115]. Studies assessing in deep the epidemiology of candidemia/IC in SOT recipients have shown a shift toward non-albicans *Candida species* over time with an increasing prevalence of *N. glabratus* and *C. parapsilosis* [116, 117], species associated with reduced susceptibility to azoles. *A. fumigatus *sensu* strictu* is the prevalent cause of IA in SOT recipients, with *A. terreus* and *A. flavus* representing less than 20% of isolates [118]. Azole resistance is an emerging issue in IA, mainly after SOT [44]. It has been associated with the isolation of *Aspergillus* cryptic species or with the selection of azole-resistant-*A. fumigatus* mediated or not by non-environment associated mutations and linked or not with prolonged exposure to azoles [119, 120]. Usually, IFI occurs within the first 6 months after transplantation; however, delayed episodes are also observed. A complicated post-transplant course is generally associated with early IFI, while persistent profound immunosuppression is the main predisposing factor for late IFI [121]. Specific risk factors for IC and for IA have been described in each type of SOT (i.e., high-MELD and choledocojejunostomy for liver transplantation; single lung and bronchial stent or ischemia for lung transplantation) [122]. A recent metanalysis aimed at identifying risk factors for IFI within the first year after SOT, showed reoperation, post-transplant renal replacement therapy (RRT), and Cytomegalovirus disease as having a high certainty of evidence and strong associations (relative effect estimate ≥ 2) across all types of SOT [123]. Antifungal prophylaxis is the main strategy to prevent IFI after SOT. Old studies assessing the universal prophylaxis showed a reduced incidence of IFI and IFI-associated mortality, but no impact on overall mortality, on the other hand, a shift toward non-albicans *Candida species* was observed [124]. Thus, a targeted approach is currently recommended limiting the use of antifungal prophylaxis to patients at high risk for IFI [122]. Indeed, this approach has been shown to be effective and feasible in real life [125]. However, the choice of the best antifungal agent for prophylaxis in SOT recipients at high risk of IFI is controversial [126]. One RCT including liver transplant (LT) recipients at high risk for IFI showed no difference between anidulafungin and fluconazole, but it was limited by a low rate of IFI (only 2 episodes of IA in the fluconazole group) [127]. One meta-analysis did not find a difference in preventing IFI between amphotericin B and fluconazole, but it included very old studies [128]. One propensity-matched multicenter cohort study showed no difference in the overall rate of IFI between caspofungin and fluconazole after LT. However, after adjusting for confounders, caspofungin was associated with a lower rate of IA [129]. High-risk patients receiving L-AmB as antifungal prophylaxis after LT showed the lowest risk of breakthrough IFI compared with those receiving no prophylaxis, fluconazole, or echinocandins in a multicenter cohort study [130]. An increased risk of breakthrough IFI associated with echinocandin prophylaxis after LT was also confirmed by a meta-analysis [131]. Finally, considering drug–drug interaction, the need for TDM, and safety issues, triazoles are considered not easy to handle after SOT, mainly in LT recipients. For all the above considerations, the use of pulsed doses of L-AmB is considered the better option mainly in the setting of LT. In a phase II uncontrolled trial including 76 high-risk LT recipients, prophylaxis with L-AmB administered at the dosage of 10 mg/kg once weekly was shown to be safe with only 3 patients developing acute kidney injury unrelated to the study drug; in addition, the IFI rate was significantly lower than that observed in a historical control group (2.6% vs. 11.8%, *p* = 0.03) [132]. Recommendations about the therapeutic management of IC and IA in SOT recipients are the same as for non-SOT recipients [133]. For IA, isavuconazole has been shown to be safe and effective in the management of SOT recipients with invasive mold infections [134]. Compared with voriconazole and posaconazole, isavuconazole has fewer drug-drug interactions with immunosuppressant drugs. A recent single-center retrospective cohort study including 68 patients (51 lungs, 14 hearts, and 3 heart/lung transplant recipients) investigated the concentration to dosage ratios (C/D) of immunosuppressants when starting isavuconazole de novo or shifting to isavuconazole from other azole treatment. The authors observed a temporary doubling of tacrolimus exposure, as well as a required dose decrease for cyclosporine and sirolimus when starting isavuconazole de novo. Tacrolimus C/D increased by 110% at day 3 in patients started on isavuconazole de novo. When transitioning from other azoles, tacrolimus and cyclosporine required about twice the initial dose [135]. Finally, although routine TDM of isavuconazole exposure is not routinely recommended, in patients with severe liver disease, an increased exposure may occur, thus requiring dosage adjustment [136]. L-AmB is considered the best option in patients in whom first-line therapy is associated with an unacceptable adverse-event profile, drug–drug interaction, or risk for resistant/refractory disease [133, 137].

### Statements

23. In SOT recipients, a targeted (risk-based) approach to antifungal prophylaxis is recommended. Clinically relevant drug–drug interactions, safety concerns, and rates of breakthrough infections are all issues to be taken into account when choosing an antifungal agent for prophylaxis. In this regard, L-AmB may be considered a suitable option. *
24. Drug–drug interactions with immunosuppressive drugs could sometimes represent a relevant issue when treating IFI with azole antifungals after SOT. In this regard, L-AmB could be a valuable alternative option for the empirical treatment of IFI. *
25. Regarding IC in SOT recipients, L-AmB could be considered a reasonable alternative to echinocandins. *
26. Since antifungal stewardship has emerged as an important component of quality in managing IFI, the application of a targeted prophylaxis or pre-emptive antifungal treatment is a valuable approach in every transplant setting, including lung transplant. *

#### Hematologic malignancies

### Background

Patients with long-term neutropenia following chemotherapy for acute myeloid leukemia (AML) and patients undergoing allogeneic hematopoietic stem cell transplantation (HSCT) are at high risk of contracting IFIs. Moreover, new risk categories are emerging, for example, patients treated with immunotherapy or chimeric antigen receptor (CAR) T cell therapy who may develop prolonged phases of severe neutropenia during and following the treatment [138]. The clinical efficacy of antifungal prophylaxis in high-risk patients has been demonstrated in randomized controlled trials and is now recommended in international guidelines [22, 139–142]. Although this strategy has resulted in a decline in the incidence of IFIs in high-risk hematology patients, a subset of such patients still develops breakthrough IFIs (bIFIs) [143]. Cohort studies conducted after the introduction of posaconazole as the standard of care for prophylaxis in this setting further highlighted the development of posaconazole-associated bIFIs with variable incidence rates depending on the study (0–10.9%) [144]. IA caused by *A. fumigatus* is most often represented among these bIFIs, but IA caused by non-*fumigatus* species and bIFIs caused by non-*Aspergillus* molds have also been reported including several cases of mucormycosis and fusariosis [12, 145–150]. The occurrence of bIFI in this setting may be explained by three clinical scenarios, in addition to a severe immune deficit or increased fungal virulence [143]: (i) sub-therapeutic drug levels in patients receiving azole prophylaxis, (ii) azole-resistant *Aspergillus fumigatus*, and (iii) intrinsic posaconazole-resistant IFI (some *Mucorales* strains, *Fusarium*, or some other rare molds). In these scenarios, the choice of treatment should be individualized according to several factors, but in most cases, the initiation of treatment with L-AmB is appropriate as this drug provides broad-spectrum coverage against azole-susceptible and azole-resistant *Aspergillus,* various species of *Mucorales*, *Fusarium*, some—but not all—other filamentous fungi and common or rare yeasts [13, 23]. The treatment should be continued based on antifungal susceptibility testing results, if available.

Although there are several new antifungal agents in the pipeline, triazoles continue to be the mainstay of therapy for the treatment and prevention of IFIs in hematological patients, but their clinical use is complicated by variable pharmacokinetics and drug–drug interactions. Therefore, there is increased recognition of the need for antifungal stewardship and practical guidance for TDM for patients with IFIs.

Given the marked intra- and inter-patient pharmacokinetic variability of voriconazole and the association of plasma exposure with both efficacy and toxicity, voriconazole concentrations should be routinely monitored in patients receiving this agent for prophylaxis or treatment [151, 152]. As previously reported, even if it was generally accepted that isavuconazole has lower variability in terms of pharmacokinetics, recent studies suggest that, especially in the ICU setting, isavuconazole plasma concentrations may vary in critically ill patients and significantly lower isavuconazole levels were observed in patients with elevated body mass index and higher SOFA score [153–155]. Overall, these studies indicate that TDM for azole is strictly necessary in the ICU setting to optimize efficacy and reduce unintended side effects [156]. Therefore, in centers where TDM is not available an alternative treatment to azole such as L-AmB could be considered when treating a critically ill patient with suspected or confirmed invasive mold infection.

Considering the high risk of IFI in certain hematology patients, such as those with prolonged neutropenia or after allogeneic HSCT, and a wide spectrum of fungal pathogens, pre-emptive therapy L-AmB, which is fungicidal against both yeasts and molds, can be useful in ICU-admitted patients with clinical suspicion of IFIs based on one of the following: radiological findings, or cultures from non-sterile, mainly respiratory, materials, or non-culture based tests, such as GM or PCR [157]. A complete diagnostic work-up should be performed, including sampling at the site of infection, and antifungal treatment should be discontinued if the suspicion of IFI is not confirmed.

### Statements

27. Antifungal prophylaxis, either with fluconazole to target *Candida species* or with posaconazole to target also molds, is recommended only in some selected high-risk populations of hematology patients (e.g., a mold-active agent in case of neutropenic patients undergoing induction chemotherapy for AML or allogeneic HSCT, or patients with graft-versus-host disease; fluconazole for patients receiving high-dose chemotherapy for aggressive lymphoma).
28. Patients with hematologic malignancies receiving mold-active azole prophylaxis who develop suspected or documented breakthrough IFI should receive treatment with L-AmB and promptly undergo a complete diagnostic workup.
29. Patients with hematologic malignancies admitted to the ICU and having IFI with no possibility for TDM of azoles and/or at high risk of azole-related drug–drug interactions should receive treatment with L-AmB.
30. Considering the high risk of IFI and wide spectrum of fungal pathogens in certain hematology patients (with prolonged neutropenia or after allogeneic HSCT), empirical therapy with L-AmB can be useful in patients admitted in ICU with clinical suspicion of IFIs while completing diagnostic work-up and it should be discontinued if the suspicion of IFI is not confirmed. *

#### Abdominal surgery

### Background

Intra-abdominal candidiasis is the most common type of deep-seated candidiasis [158]. Although *Candida* invasion and dissemination within the abdominal cavity may occur, IAC is rarely accompanied by candidemia [90]. Thus, diagnosis of IAC without bloodstream infection may be difficult, especially in the absence of a non-culture-based gold standard method [159].

Because of the poor prognosis of IC in critically ill patients, empirical antifungal treatment is commonly administrated. However, less than 10% of ICU patients receiving an empirical antifungal therapy for suspected IC obtain a microbiological diagnosis [160]. To identify ICU patients who may benefit from the early introduction of antifungal therapy, some strategies based on clinical characteristics have been proposed. For instance, a recent algorithm differentiated patients based on the presence of septic shock [161]. Other prediction rules based the choice on the assessment of multifocal Candida [37, 162]. Despite these scores being suitable for patient bedside evaluation, they may overestimate the risk IC brings to the extensive use of antifungals.

The choice of drug for IAC is another critical issue. Currently, guidelines recommend echinocandins as the first-line treatment for IC. However, recent literature suggests their intra-abdominal penetration is limited [163–165]. The high plasma protein binding (> 95%) significantly affects their passive diffusion into the peritoneal fluid [65, 166]; indeed, only the unbound fraction passes from the vascular to the extravascular compartment. It is estimated that only 33% of the echinocandin dose reaches the intra-abdominal cavity [167]; moreover, some PK studies documented a low probability of PK/PD target attainment using standard dosing regimens, especially for less susceptible Candida species [149, 152]. For these reasons, some authors proposed to use of higher dosages of echinocandins for the treatment of IAC, but definitive data are lacking [168, 169]. In other terms, abdominal candidiasis could represent a hidden reservoir of resistance to echinocandins with a higher risk of failure despite adequate source control [134, 159].

L-AmB has good activity against *Candida species*, a low potential for inducing resistance, concentration-dependent fungicidal activity, a prolonged post-antifungal effect, and a potent anti-biofilm effect. Unlike echinocandins, available PK data evidenced that, unlike echinocandins, L-AmB did not show any significant difference in concentrations between healthy volunteers and critical patients; moreover, no decrease in Cmax or AUC was observed in patients undergoing CRRT [159]. The efficacy of L-AmB increases linearly with its concentration, showing strong fungicidal activity in deep-seated compartments such as the pleura, peritoneum, pericardium, aqueous humor, and vitreous [163]. A recently published therapeutic decision algorithm placed L-AmB as a first-line treatment in suspected or confirmed cases of IAC and sepsis/septic shock with candidemia or endophthalmitis as well as IAC and sepsis/septic shock with previous exposition to echinocandins and/or fluconazole or risk factors for *N. glabratus* infection [159]. In the case of echinocandin-resistant *C. auris* use of L-AmB, 5 mg/kg/day was proposed alone or in combination with echinocandins, as in vitro synergic activity was demonstrated [159, 170, 171].

Of course, along with antifungal therapy, an appropriate source control remains a key component of the treatment of critically ill surgical patients with IAC [172].

### Statements

31. Diagnosis of IAC remains challenging. It is based on microscopy and culture of specimens obtained during surgery or by percutaneous aspiration. Blood cultures must be taken but might not be helpful for diagnosis due to lack of sensitivity. Non-culturable methods, BDG determination, or other tools might be used to exclude fungal etiology.
32. In patients with IAC, the choice of empirical antifungal therapy should be guided by host, microbiological, and epidemiological variables. L-AmB could be considered first-line therapy in cases of IAC with sepsis/septic shock, the risk for *N. glabratus* and *C. parapsilosis* infections, or previous therapy with echinocandins. *
33. Echinocandins could be used as a first-choice treatment in non-critically ill patients. However, recent pharmacokinetics/pharmacodynamics evidence suggested that exposure to the ascitic fluid may be suboptimal and may cause breakthrough resistance, especially in the case of non-albicans etiology. *
34. Combination therapy with L-AmB and an echinocandin should be considered a rescue therapy in the case of *C. auris* etiology.
35. In critically ill patients, empirical antifungal therapy for suspected IC (including those with potential abdominal origin) may be safely interrupted early according to a biomarker-driven strategy. *

## Conclusions

Treatment of IFI in critical care is still challenging due to the growing number of patients at risk and the emergence of drug-resistant fungal species. With its broad-spectrum activity and major safety compared with previous formulations, LAMB may represent a suitable therapeutic choice for many clinical scenarios. For this reason, a multidisciplinary panel of 16 Italian experts developed 35 statements on the use of LAMB in ICU based on a scoping review of the most updated literature. Though the scientific debate on the place in therapy of LAMB is ongoing, this consensus document would first reach out to unmet clinical needs in critical care. Differently from current guidelines, this paper uncovers common clinical situations where LAMB may be a front-line therapy, consequently encouraging a more appropriate use.

This study has some limitations. First, this document is based on expert opinions, as evidence on the use of LAMB in the ICU population is limited. All the panel members work in Italian centers, narrowing the scope of the contents. Indeed, the use of LAMB may be precluded by the economic charge and the unavailability in some centers. Finally, new antifungals will be available in clinical practice in a short time, broadening the therapeutic armamentarium for difficult-to-treat fungal infections.

This expert opinion paper could represent a practical tool for physicians involved in the care of critically ill patients at risk for severe fungal infections. Enhancing clinical evidence on the use of LAMB in the ICU may encourage the design of high-quality prospective studies on LAMB to improve the management of IFI in the ICU.

## Data Availability

No datasets were generated or analysed during the current study.

## References

[1] Limper AH, Knox KS, Sarosi GA, Ampel NM, Bennett JE, Catanzaro A et al (2011) An official American Thoracic Society statement: treatment of fungal infections in adult pulmonary and critical care patients. Am J Respir Crit Care Med 183:96–128. 10.1164/rccm.2008-740ST 21193785 10.1164/rccm.2008-740ST

[2] Kett DH, Azoulay E, Echeverria PM, Vincent J-L (2011) Candida bloodstream infections in intensive care units: analysis of the extended prevalence of infection in intensive care unit study*. Crit Care Med 39:665–670. 10.1097/CCM.0b013e318206c1ca 21169817 10.1097/CCM.0b013e318206c1ca

[3] Muskett H, Shahin J, Eyres G, Harvey S, Rowan K, Harrison D (2011) Risk factors for invasive fungal disease in critically ill adult patients: a systematic review. Crit Care 15:15. 10.1186/cc10574 10.1186/cc10574 PMC3388661 22126425

[4] Colombo AL, de Almeida Júnior JN, Slavin MA, Chen SCA, Sorrell TC (2017) Candida and invasive mould diseases in non-neutropenic critically ill patients and patients with haematological cancer. Lancet Infect Dis 17:e344–e356. 10.1016/S1473-3099(17)30304-3 28774702 10.1016/S1473-3099(17)30304-3

[5] Guinea J (2014) Global trends in the distribution of Candida species causing candidemia. Clin Microbiol Infect 20:5–10. 10.1111/1469-0691.12539 24506442 10.1111/1469-0691.12539

[6] Franconi I, Rizzato C, Tavanti A, Falcone M, Lupetti A (2023) Paradigm shift: Candida parapsilosis sensu stricto as the most prevalent candida species isolated from bloodstream infections with increasing azole-non-susceptibility rates: trends from 2015–2022 survey. J Fungi 9:1012. 10.3390/jof9101012 10.3390/jof9101012 PMC10608153 37888268

[7] Chi H-W, Yang Y-S, Shang S-T, Chen K-H, Yeh K-M, Chang F-Y et al (2011) Candida albicans versus non-albicans bloodstream infections: the comparison of risk factors and outcome. J Microbiol Immunol Infect 44:369–375. 10.1016/j.jmii.2010.08.010 21524971 10.1016/j.jmii.2010.08.010

[8] Taccone FS, Van den Abeele A-M, Bulpa P, Misset B, Meersseman W, Cardoso T et al (2015) Epidemiology of invasive aspergillosis in critically ill patients: clinical presentation, underlying conditions, and outcomes. Crit Care 19:7. 10.1186/s13054-014-0722-7 25928694 10.1186/s13054-014-0722-7 PMC4344741

[9] Schauwvlieghe AFAD, Rijnders BJA, Philips N, Verwijs R, Vanderbeke L, Van Tienen C et al (2018) Invasive aspergillosis in patients admitted to the intensive care unit with severe influenza: a retrospective cohort study. Lancet Respir Med 6:782–792. 10.1016/S2213-2600(18)30274-1 30076119 10.1016/S2213-2600(18)30274-1

[10] Albrich WC, Lamoth F (2023) Viral-associated pulmonary aspergillosis: have we finally overcome the debate of colonization versus infection? Am J Respir Crit Care Med 208:230–231. 10.1164/rccm.202306-1022ED 37348113 10.1164/rccm.202306-1022ED PMC10395713

[11] Lass-Flörl C, Steixner S (2023) The changing epidemiology of fungal infections. Mol Aspects Med 94:101215. 10.1016/j.mam.2023.101215 37804792 10.1016/j.mam.2023.101215

[12] Lamoth F, Chung SJ, Damonti L, Alexander BD (2017) Changing epidemiology of invasive mold infections in patients receiving azole prophylaxis. Clin Infect Dis 64:1619–1621. 10.1093/cid/cix130 28199491 10.1093/cid/cix130

[13] Hoenigl M, Salmanton-García J, Walsh TJ, Nucci M, Neoh CF, Jenks JD et al (2021) Global guideline for the diagnosis and management of rare mould infections: an initiative of the European Confederation of Medical Mycology in cooperation with the International Society for Human and Animal Mycology and the American Society for Microbiology. Lancet Infect Dis 21:e246–e257. 10.1016/S1473-3099(20)30784-2 33606997 10.1016/S1473-3099(20)30784-2

[14] Daneshnia F, de Almeida Júnior JN, Ilkit M, Lombardi L, Perry AM, Gao M et al (2023) Worldwide emergence of fluconazole-resistant Candida parapsilosis: current framework and future research roadmap. Lancet Microbe 4:e470–e480. 10.1016/S2666-5247(23)00067-8 37121240 10.1016/S2666-5247(23)00067-8 PMC10634418

[15] Belkin A, Gazit Z, Keller N, Ben-Ami R, Wieder-Finesod A, Novikov A et al (2018) *Candida auris* infection leading to nosocomial transmission, Israel, 2017. Emerg Infect Dis 24:801–804. 10.3201/eid2404.171715 29553329 10.3201/eid2404.171715 PMC5875262

[16] Aldejohann AM, Wiese-Posselt M, Gastmeier P, Kurzai O (2022) Expert recommendations for prevention and management of Candida auris transmission. Mycoses 65:590–598. 10.1111/myc.13445 35437832 10.1111/myc.13445

[17] van Paassen J, Russcher A, in ’t Veld - van Wingerden AW, Verweij PE, Kuijper EJ (2016) Emerging aspergillosis by azole-resistant Aspergillus fumigatus at an intensive care unit in the Netherlands, (2010) to 2013. Eurosurveillance 21. 10.2807/1560-7917.ES.2016.21.30.30300 10.2807/1560-7917.ES.2016.21.30.30300 27541498

[18] Kriegl L, Egger M, Boyer J, Hoenigl M, Krause R (2024) New treatment options for critically important WHO fungal priority pathogens. Clin Microbiol Infect. 10.1016/j.cmi.2024.03.006 38461942 10.1016/j.cmi.2024.03.006

[19] Stone NRH, Bicanic T, Salim R, Hope W (2016) Liposomal Amphotericin B (AmBisome®): a review of the pharmacokinetics, pharmacodynamics, clinical experience and future directions. Drugs 76:485–500. 10.1007/s40265-016-0538-7 26818726 10.1007/s40265-016-0538-7 PMC4856207

[20] Hoenigl M, Lewis R, van de Veerdonk FL, Verweij PE, Cornely OA (2022) Liposomal amphotericin B—the future. J Antimicrob Chemother 77:ii21-34. 10.1093/jac/dkac353 36426674 10.1093/jac/dkac353 PMC9693803

[21] Botero Aguirre JP, Restrepo Hamid AM (2015) Amphotericin B deoxycholate versus liposomal amphotericin B: effects on kidney function. Cochrane Database Syst Rev 2015. 10.1002/14651858.CD010481.pub2 10.1002/14651858.CD010481.pub2 PMC10542271 26595825

[22] Freifeld AG, Bow EJ, Sepkowitz KA, Boeckh MJ, Ito JI, Mullen CA et al (2011) Clinical practice guideline for the use of antimicrobial agents in neutropenic patients with cancer: 2010 update by the Infectious Diseases Society of America. Clin Infect Dis 52:e56-93. 10.1093/cid/cir073 21258094 10.1093/cid/cir073

[23] Chen SCA, Perfect J, Colombo AL, Cornely OA, Groll AH, Seidel D et al (2021) Global guideline for the diagnosis and management of rare yeast infections: an initiative of the ECMM in cooperation with ISHAM and ASM. Lancet Infect Dis 21:e375–e386. 10.1016/S1473-3099(21)00203-6 34419208 10.1016/S1473-3099(21)00203-6

[24] De Pascale G, Tumbarello M (2015) Fungal infections in the ICU: advances in treatment and diagnosis. Curr Opin Crit Care 21:421–429. 10.1097/MCC.0000000000000230 26165502 10.1097/MCC.0000000000000230

[25] Berkow EL, Lockhart SR, Ostrosky-Zeichner L (2020) Antifungal susceptibility testing: current approaches. Clin Microbiol Rev 33:33. 10.1128/CMR.00069-19 10.1128/CMR.00069-19 PMC7194854 32349998

[26] Jensen RH, Johansen HK, Søes LM, Lemming LE, Rosenvinge FS, Nielsen L et al (2016) Posttreatment antifungal resistance among colonizing Candida isolates in Candidemia patients: results from a systematic multicenter study. Antimicrob Agents Chemother 60:1500–1508. 10.1128/AAC.01763-15 10.1128/AAC.01763-15 PMC4775986 26711776

[27] Vallabhaneni S, Cleveland AA, Farley MM, Harrison LH, Schaffner W, Beldavs ZG, et al. (2015) Epidemiology and risk factors for echinocandin nonsusceptible Candida glabrata bloodstream infections: data from a large multisite population-based candidemia surveillance program, 2008–2014. Open Forum Infect Dis 2. 10.1093/ofid/ofv163 10.1093/ofid/ofv163 PMC4677623 26677456

[28] Eyre DW, Sheppard AE, Madder H, Moir I, Moroney R, Quan TP et al (2018) A *Candida auris* outbreak and its control in an intensive care setting. N Engl J Med 379:1322–1331. 10.1056/NEJMoa1714373 30281988 10.1056/NEJMoa1714373

[29] Alves J, Alonso-Tarrés C, Rello J (2022) How to identify invasive candidemia in ICU—a narrative review. Antibiotics 11:1804. 10.3390/antibiotics11121804 36551461 10.3390/antibiotics11121804 PMC9774599

[30] Chamilos G, Kontoyiannis DP (2006) Defining the diagnosis of invasive aspergillosis. Med Mycol 44:163–172. 10.1080/13693780600823258 10.1080/13693780600823258 30408899

[31] Posteraro B, De Pascale G, Tumbarello M, Torelli R, Pennisi M, Bello G et al (2011) Early diagnosis of candidemia in intensive care unit patients with sepsis: a prospective comparison of (1→3)-β-D-glucan assay, Candida score, and colonization index. Crit Care 15:R249. 10.1186/cc10507 22018278 10.1186/cc10507 PMC3334800

[32] Bougnoux ME, Kac G, Aegerter P, D’Enfert C, Fagon JY, Amrein C et al (2008) Candidemia and candiduria in critically ill patients admitted to intensive care units in France: incidence, molecular diversity, management and outcome. Intensive Care Med 34:292–299. 10.1007/s00134-007-0865-y 17909746 10.1007/s00134-007-0865-y

[33] Christner M, Abdennadher B, Wichmann D, Kluge S, Pepić A, Aepfelbacher M et al (2024) The added value of (1,3)-β-D-glucan for the diagnosis of Invasive Candidiasis in ICU patients: a prospective cohort study. Infection 52:73–81. 10.1007/s15010-023-02053-4 37322388 10.1007/s15010-023-02053-4 PMC10811116

[34] De Pascale G, Posteraro B, D’Arrigo S, Spinazzola G, Gaspari R, Bello G et al (2020) (1,3)-β-d-Glucan-based empirical antifungal interruption in suspected invasive candidiasis: a randomized trial. Crit Care 24:24. 10.1186/s13054-020-03265-y 32891170 10.1186/s13054-020-03265-y PMC7487510

[35] Esteves P, Lopes Lima S, de Azevedo Salles, Melo A, Maria Beirão E, Nucci M, Colombo AL (2021) (1,3)-β-D-glucan is able to predict therapeutic failure of patients with candidemia and not only mortality. Mycoses 64:264–271. 10.1111/myc.13224 33274533 10.1111/myc.13224

[36] Duettmann W, Koidl C, Krause R, Lackner G, Woelfler A, Hoenigl M (2016) Specificity of mannan antigen and anti-mannan antibody screening in patients with haematological malignancies at risk for fungal infection. Mycoses 59:374–378. 10.1111/myc.12482 26916753 10.1111/myc.12482

[37] León C, Ruiz-Santana S, Saavedra P, Castro C, Úbeda A, Loza A et al (2012) Value of β-d-glucan and Candida albicans germ tube antibody for discriminating between Candida colonization and invasive candidiasis in patients with severe abdominal conditions. Intensive Care Med 38:1315–1325. 10.1007/s00134-012-2616-y 22752333 10.1007/s00134-012-2616-y

[38] Pini P, Colombari B, Marchi E, Castagnoli A, Venturelli C, Sarti M et al (2019) Performance of Candida albicans germ tube antibodies (CAGTA) and its association with (1 → 3)-β-D-glucan (BDG) for diagnosis daof invasive candidiasis (IC). Diagn Microbiol Infect Dis 93:39–43. 10.1016/j.diagmicrobio.2018.07.007 30098849 10.1016/j.diagmicrobio.2018.07.007

[39] Lass-Flörl C (2019) How to make a fast diagnosis in invasive aspergillosis. Med Mycol 57:S155–S160. 10.1093/mmy/myy103 30816965 10.1093/mmy/myy103

[40] Clancy C, Nguyen MH (2018) Non-culture diagnostics for invasive Candidiasis: promise and unintended consequences. J Fungi 4:27. 10.3390/jof4010027 10.3390/jof4010027 PMC5872330 29463043

[41] Bloos F, Held J, Kluge S, Simon P, Kogelmann K, de Heer G et al (2022) (1 → 3)-β-d-Glucan-guided antifungal therapy in adults with sepsis: the CandiSep randomized clinical trial. Intensive Care Med 48:865–875. 10.1007/s00134-022-06733-x 35708758 10.1007/s00134-022-06733-x PMC9273538

[42] Patterson TF, Thompson GR, Denning DW, Fishman JA, Hadley S, Herbrecht R et al (2016) Practice guidelines for the diagnosis and management of Aspergillosis: 2016 update by the Infectious Diseases Society of America. Clin Infect Dis 63:e1-60. 10.1093/cid/ciw326 27365388 10.1093/cid/ciw326 PMC4967602

[43] Mercier T, Guldentops E, Lagrou K, Maertens J (2018) Galactomannan, a surrogate marker for outcome in invasive aspergillosis: finally coming of age. Front Microbiol 9:9. 10.3389/fmicb.2018.00661 29670608 10.3389/fmicb.2018.00661 PMC5893815

[44] Baddley JW, Marr KA, Andes DR, Walsh TJ, Kauffman CA, Kontoyiannis DP et al (2009) Patterns of susceptibility of *Aspergillus* isolates recovered from patients enrolled in the transplant-associated infection surveillance network. J Clin Microbiol 47:3271–3275. 10.1128/JCM.00854-09 19692558 10.1128/JCM.00854-09 PMC2756905

[45] Mikulska M, Furfaro E, Dettori S, Giacobbe DR, Magnasco L, Dentone C et al (2022) *Aspergillus* -PCR in bronchoalveolar lavage - diagnostic accuracy for invasive pulmonary aspergillosis in critically ill patients. Mycoses 65:411–418. 10.1111/myc.13428 35138675 10.1111/myc.13428

[46] Krifors A, Özenci V, Ullberg M, Ackefors M, Jädersten M, Strålin K et al (2019) PCR with electrospray ionization-mass spectrometry on bronchoalveolar lavage for detection of invasive mold infections in hematological patients. PLoS ONE 14:e0212812. 10.1371/journal.pone.0212812 30794675 10.1371/journal.pone.0212812 PMC6386253

[47] Huygens S, Dunbar A, Buil JB, Klaassen CHW, Verweij PE, van Dijk K et al (2023) clinical impact of polymerase chain reaction–based *Aspergillus* and azole resistance detection in invasive aspergillosis: a prospective multicenter study. Clin Infect Dis 77:38–45. 10.1093/cid/ciad141 36905147 10.1093/cid/ciad141 PMC10320047

[48] Calandra T, Roberts JA, Antonelli M, Bassetti M, Vincent JL (2016) Diagnosis and management of invasive candidiasis in the ICU: an updated approach to an old enemy. Crit Care 20:20. 10.1186/s13054-016-1313-6 27230564 10.1186/s13054-016-1313-6 PMC4882871

[49] Haydour Q, Hage CA, Carmona EM, Epelbaum O, Evans SE, Gabe LM et al (2019) Diagnosis of fungal infections. A systematic review and meta-analysis supporting American Thoracic Society Practice Guideline. Ann Am Thorac Soc 16:1179–1188. 10.1513/AnnalsATS.201811-766OC 31219341 10.1513/AnnalsATS.201811-766OC

[50] Tragiannidis A, Linke C, Correa-Martinez CL, Herbrüggen H, Schaumburg F, Groll AH (2023) Long-term kinetics of serum galactomannan during treatment of complicated invasive pulmonary aspergillosis. J Fungi 9:157. 10.3390/jof9020157 10.3390/jof9020157 PMC9965572 36836274

[51] Kovanda LL, Desai AV, Hope WW (2017) Prognostic value of galactomannan: current evidence for monitoring response to antifungal therapy in patients with invasive aspergillosis. J Pharmacokinet Pharmacodyn 44:143–151. 10.1007/s10928-017-9509-1 28181136 10.1007/s10928-017-9509-1

[52] Eigl S, Prattes J, Lackner M, Willinger B, Spiess B, Reinwald M et al (2015) Multicenter evaluation of a lateral-flow device test for diagnosing invasive pulmonary aspergillosis in ICU patients. Crit Care 19:178. 10.1186/s13054-015-0905-x 25927915 10.1186/s13054-015-0905-x PMC4421996

[53] Fang W, Wu J, Cheng M, Zhu X, Du M, Chen C et al (2023) Diagnosis of invasive fungal infections: challenges and recent developments. J Biomed Sci 30:42. 10.1186/s12929-023-00926-2 37337179 10.1186/s12929-023-00926-2 PMC10278348

[54] Han Y, Wu X, Jiang G, Guo A, Jin Z, Ying Y et al (2023) Bronchoalveolar lavage fluid polymerase chain reaction for invasive pulmonary aspergillosis among high-risk patients: a diagnostic meta-analysis. BMC Pulm Med 23:58. 10.1186/s12890-023-02343-5 36750828 10.1186/s12890-023-02343-5 PMC9906844

[55] Ullmann AJ, Aguado JM, Arikan-Akdagli S, Denning DW, Groll AH, Lagrou K et al (2018) Diagnosis and management of Aspergillus diseases: executive summary of the 2017 ESCMID-ECMM-ERS guideline. Clin Microbiol Infect 24:e1-38. 10.1016/j.cmi.2018.01.002 29544767 10.1016/j.cmi.2018.01.002

[56] Sugawara Y, Nakase K, Nakamura A, Ohishi K, Sugimoto Y, Fujieda A et al (2013) Clinical utility of a panfungal polymerase chain reaction assay for invasive fungal diseases in patients with haematologic disorders. Eur J Haematol 90:331–339. 10.1111/ejh.12078 23360173 10.1111/ejh.12078

[57] Mandhaniya S, Iqbal S, Sharawat SK, Xess I, Bakhshi S (2012) Diagnosis of invasive fungal infections using real-time PCR assay in paediatric acute leukaemia induction. Mycoses 55:372–379. 10.1111/j.1439-0507.2011.02157.x 22420668 10.1111/j.1439-0507.2011.02157.x

[58] Mercier T, Guldentops E, Patteet S, Beuselinck K, Lagrou K, Maertens J (2019) Beta- d -glucan for diagnosing pneumocystis pneumonia: a direct comparison between the Wako β-glucan assay and the fungitell assay. J Clin Microbiol 57:57. 10.1128/JCM.00322-19 10.1128/JCM.00322-19 PMC6535610 30918045

[59] Temfack E, Rim JJB, Spijker R, Loyse A, Chiller T, Pappas PG et al (2021) Cryptococcal antigen in serum and cerebrospinal fluid for detecting cryptococcal meningitis in adults living with human immunodeficiency virus: systematic review and meta-analysis of diagnostic test accuracy studies. Clin Infect Dis 72:1268–1278. 10.1093/cid/ciaa1243 32829406 10.1093/cid/ciaa1243 PMC8522332

[60] Miwa T, Okamoto K, Ikeuchi K, Yamamoto S, Okugawa S, Ichida A et al (2024) The role of frequent screening or diagnostic testing of serum cryptococcal antigen in liver transplant recipients: a descriptive epidemiology. Open Forum Infect Dis 11:ofae255. 10.1093/ofid/ofae255 38774792 10.1093/ofid/ofae255 PMC11108085

[61] Hage CA, Ribes JA, Wengenack NL, Baddour LM, Assi M, McKinsey DS et al (2011) A multicenter evaluation of tests for diagnosis of histoplasmosis. Clin Infect Dis 53:448–454. 10.1093/cid/cir435 21810734 10.1093/cid/cir435

[62] Senchyna F, Hogan CA, Murugesan K, Moreno A, Ho DY, Subramanian A et al (2021) Clinical accuracy and impact of plasma cell-free DNA fungal polymerase chain reaction panel for noninvasive diagnosis of fungal infection. Clin Infect Dis 73:1677–1684. 10.1093/cid/ciab158 33606010 10.1093/cid/ciab158

[63] Van Daele R, Wauters J, Elkayal O, Dreesen E, Debaveye Y, Lagrou K et al (2022) Liposomal amphotericin B exposure in critically ill patients: a prospective pharmacokinetic study. Med Mycol 60:60. 10.1093/mmy/myac074 10.1093/mmy/myac074 36124725

[64] Heinemann V, Bosse D, Jehn U, Kähny B, Wachholz K, Debus A et al (1997) Pharmacokinetics of liposomal amphotericin B (Ambisome) in critically ill patients. Antimicrob Agents Chemother 41:1275–1280. 10.1128/AAC.41.6.1275 9174183 10.1128/aac.41.6.1275 PMC163899

[65] Bellmann R, Smuszkiewicz P (2017) Pharmacokinetics of antifungal drugs: practical implications for optimized treatment of patients. Infection 45:737–779. 10.1007/s15010-017-1042-z 28702763 10.1007/s15010-017-1042-z PMC5696449

[66] Sato S, Kamata W, Fukaguchi K, Tsunoda S, Kamio T, Koyama H et al (2022) Successful treatment of invasive tracheobronchial pulmonary aspergillosis with venovenous extracorporeal membrane oxygenation and combined systemic, intratracheal instillation of liposomal amphotericin B: a case report. J Med Case Rep 16:470. 10.1186/s13256-022-03692-1 36536458 10.1186/s13256-022-03692-1 PMC9764550

[67] Zhao Y, Seelhammer TG, Barreto EF, Wilson JW (2020) Altered pharmacokinetics and dosing of liposomal amphotericin B and isavuconazole during extracorporeal membrane oxygenation. Pharmacotherapy: J Hum Pharmacol Drug Ther 40:89–95. 10.1002/phar.2348 10.1002/phar.2348 31742741

[68] Branick K, Taylor MJ, Trump MW, Wall GC (2019) Apparent interference with extracorporeal membrane oxygenation by liposomal amphotericin B in a patient with disseminated blastomycosis receiving continuous renal replacement therapy. Am J Health Syst Pharm 76:810–813. 10.1093/ajhp/zxz054 30994894 10.1093/ajhp/zxz054

[69] Lyster H, Shekar K, Watt K, Reed A, Roberts JA, Abdul-Aziz M-H (2023) Antifungal dosing in critically ill patients on extracorporeal membrane oxygenation. Clin Pharmacokinet 62:931–942. 10.1007/s40262-023-01264-0 37300631 10.1007/s40262-023-01264-0 PMC10338597

[70] Ting MH, Spec A, Micek ST, Ritchie DJ, Krekel T (2021) Evaluation of total body weight versus adjusted body weight liposomal amphotericin b dosing in obese patients. Antimicrob Agents Chemother 65:65. 10.1128/AAC.02366-20 10.1128/AAC.02366-20 PMC8370224 34125591

[71] Nix DE, Hayes JF, Al Obaidi M, Zangeneh T (2021) Fixed dosing of amphotericin B in morbidly obese individuals. Clin Infect Dis 72:e431–e431. 10.1093/cid/ciaa1076 32725197 10.1093/cid/ciaa1076

[72] Wasmann RE, Smit C, van Dongen EPH, Wiezer RMJ, Adler-Moore J, de Beer YM et al (2020) Fixed dosing of liposomal amphotericin B in morbidly obese individuals. Clin Infect Dis 70:2213–2215. 10.1093/cid/ciz885 31588493 10.1093/cid/ciz885 PMC7201409

[73] Fu X, Zhang C, Lin X, Zheng X, Liu Q, Jin Y. Safety and effectiveness of high-dose liposomal amphotericin B: a systematic review and meta-analysis. Altern Ther Health Med 2023. 37971463

[74] Jarvis JN, Lawrence DS, Meya DB, Kagimu E, Kasibante J, Mpoza E et al (2022) Single-dose liposomal amphotericin b treatment for cryptococcal meningitis. N Engl J Med 386:1109–1120. 10.1056/NEJMoa2111904 35320642 10.1056/NEJMoa2111904 PMC7612678

[75] Ruhnke M, Cornely OA, Schmidt-Hieber M, Alakel N, Boell B, Buchheidt D et al (2020) Treatment of invasive fungal diseases in cancer patients—revised 2019 Recommendations of the Infectious Diseases Working Party (AGIHO) of the German Society of Hematology and Oncology (DGHO). Mycoses 63:653–682. 10.1111/myc.13082 32236989 10.1111/myc.13082

[76] Ekram MdR, Amin MR, Hasan MJ, Khan MdAS, Nath R, Mallik PK et al (2021) Efficacy and safety of single-dose liposomal amphotericin B in patients with visceral leishmaniasis in Bangladesh: a real-life experience. J Parasit Dis 45:903–911. 10.1007/s12639-021-01379-w 34789971 10.1007/s12639-021-01379-w PMC8556465

[77] Dondi A, Manieri E, Gambuti G, Varani S, Campoli C, Zama D et al (2023) A 10-year retrospective study on pediatric visceral leishmaniasis in a European endemic area: diagnostic and short-course therapeutic strategies. Healthcare 12:23. 10.3390/healthcare12010023 38200929 10.3390/healthcare12010023 PMC10779246

[78] Rinaldi M, Bartoletti M, Bonazzetti C, Caroccia N, Gatti M, Tazza B et al (2023) Tolerability of pulsed high-dose L-AmB as pre-emptive therapy in patients at high risk for intra-abdominal candidiasis: a phase 2 study (LAMBDA study). Int J Antimicrob Agents 62:106998. 10.1016/j.ijantimicag.2023.106998 37838147 10.1016/j.ijantimicag.2023.106998

[79] Obata Y, Takazono T, Tashiro M, Ota Y, Wakamura T, Takahashi A et al (2021) The clinical usage of liposomal amphotericin B in patients receiving renal replacement therapy in Japan: a nationwide observational study. Clin Exp Nephrol 25:279–287. 10.1007/s10157-020-01989-3 33179180 10.1007/s10157-020-01989-3 PMC7925490

[80] Feys S, Gonçalves SM, Khan M, Choi S, Boeckx B, Chatelain D et al (2022) Lung epithelial and myeloid innate immunity in influenza-associated or COVID-19-associated pulmonary aspergillosis: an observational study. Lancet Respir Med 10:1147–1159. 10.1016/S2213-2600(22)00259-4 36029799 10.1016/S2213-2600(22)00259-4 PMC9401975

[81] Salazar F, Bignell E, Brown GD, Cook PC, Warris A (2022) pathogenesis of respiratory viral and fungal coinfections. Clin Microbiol Rev 35. 10.1128/CMR.00094-21 10.1128/CMR.00094-21 PMC8597983 34788127

[82] Vanderbeke L, Jacobs C, Feys S, Reséndiz-Sharpe A, Debaveye Y, Hermans G et al (2023) A pathology-based case series of influenza- and COVID-19–associated pulmonary aspergillosis: the proof is in the tissue. Am J Respir Crit Care Med 208:301–311. 10.1164/rccm.202208-1570OC 37311243 10.1164/rccm.202208-1570OC PMC10395719

[83] Koehler P, Bassetti M, Chakrabarti A, Chen SCA, Colombo AL, Hoenigl M et al (2021) Defining and managing COVID-19-associated pulmonary aspergillosis: the 2020 ECMM/ISHAM consensus criteria for research and clinical guidance. Lancet Infect Dis 21:e149–e162. 10.1016/S1473-3099(20)30847-1 33333012 10.1016/S1473-3099(20)30847-1 PMC7833078

[84] Verweij PE, Rijnders BJA, Brüggemann RJM, Azoulay E, Bassetti M, Blot S et al (2020) Review of influenza-associated pulmonary aspergillosis in ICU patients and proposal for a case definition: an expert opinion. Intensive Care Med 46:1524–1535. 10.1007/s00134-020-06091-6 32572532 10.1007/s00134-020-06091-6 PMC7306567

[85] Vanderbeke L, Janssen NAF, Bergmans DCJJ, Bourgeois M, Buil JB, Debaveye Y et al (2021) Posaconazole for prevention of invasive pulmonary aspergillosis in critically ill influenza patients (POSA-FLU): a randomised, open-label, proof-of-concept trial. Intensive Care Med 47:674–686. 10.1007/s00134-021-06431-0 34050768 10.1007/s00134-021-06431-0 PMC8164057

[86] Brunet K, Martellosio J-P, Tewes F, Marchand S, Rammaert B (2022) Inhaled antifungal agents for treatment and prophylaxis of bronchopulmonary invasive mold infections. Pharmaceutics 14:641. 10.3390/pharmaceutics14030641 35336015 10.3390/pharmaceutics14030641 PMC8949245

[87] Hawes AM, Permpalung N (2022) Diagnosis and antifungal prophylaxis for COVID-19 associated pulmonary aspergillosis. Antibiotics 11:1704. 10.3390/antibiotics11121704 36551361 10.3390/antibiotics11121704 PMC9774425

[88] Blot SI, Taccone FS, Van den Abeele A-M, Bulpa P, Meersseman W, Brusselaers N et al (2012) A clinical algorithm to diagnose invasive pulmonary aspergillosis in critically ill patients. Am J Respir Crit Care Med 186:56–64. 10.1164/rccm.201111-1978OC 22517788 10.1164/rccm.201111-1978OC

[89] Gangneux J-P, Dannaoui E, Fekkar A, Luyt C-E, Botterel F, De Prost N et al (2022) Fungal infections in mechanically ventilated patients with COVID-19 during the first wave: the French multicentre MYCOVID study. Lancet Respir Med 10:180–190. 10.1016/S2213-2600(21)00442-2 34843666 10.1016/S2213-2600(21)00442-2 PMC8626095

[90] Prattes J, Wauters J, Giacobbe DR, Salmanton-García J, Maertens J, Bourgeois M et al (2022) Risk factors and outcome of pulmonary aspergillosis in critically ill coronavirus disease 2019 patients—a multinational observational study by the European Confederation of Medical Mycology. Clin Microbiol Infect 28:580–587. 10.1016/j.cmi.2021.08.014 34454093 10.1016/j.cmi.2021.08.014 PMC8387556

[91] Otu A, Kosmidis C, Mathioudakis AG, Ibe C, Denning DW (2023) The clinical spectrum of aspergillosis in chronic obstructive pulmonary disease. Infection 51:813–829. 10.1007/s15010-022-01960-2 36662439 10.1007/s15010-022-01960-2 PMC9857914

[92] Gu Y, Ye X, Liu Y, Wang Y, Shen K, Zhong J et al (2021) A risk-predictive model for invasive pulmonary aspergillosis in patients with acute exacerbation of chronic obstructive pulmonary disease. Respir Res 22:176. 10.1186/s12931-021-01771-3 34107968 10.1186/s12931-021-01771-3 PMC8188951

[93] Chakrabarti A, Das A, Mandal J, Shivaprakash MR, George VK, Tarai B et al (2006) The rising trend of invasive zygomycosis in patients with uncontrolled diabetes mellitus. Med Mycol 44:335–342. 10.1080/13693780500464930 16772227 10.1080/13693780500464930

[94] Steinbrink JM, Miceli MH (2021) Mucormycosis. Infect Dis Clin North Am 35:435–452. 10.1016/j.idc.2021.03.009 34016285 10.1016/j.idc.2021.03.009 PMC10110349

[95] Jeong W, Keighley C, Wolfe R, Lee WL, Slavin MA, Kong DCM et al (2019) The epidemiology and clinical manifestations of mucormycosis: a systematic review and meta-analysis of case reports. Clin Microbiol Infect 25:26–34. 10.1016/j.cmi.2018.07.011 30036666 10.1016/j.cmi.2018.07.011

[96] Sengupta I, Nayak T (2022) Coincidence or reality behind Mucormycosis, diabetes mellitus and Covid-19 association: a systematic review. J Med Mycol 32:101257. 10.1016/j.mycmed.2022.101257 10.1016/j.mycmed.2022.101257 PMC8855615 35219907

[97] Cornely OA, Alastruey-Izquierdo A, Arenz D, Chen SCA, Dannaoui E, Hochhegger B et al (2019) Global guideline for the diagnosis and management of mucormycosis: an initiative of the European Confederation of Medical Mycology in cooperation with the Mycoses Study Group Education and Research Consortium. Lancet Infect Dis 19:e405–e421. 10.1016/S1473-3099(19)30312-3 31699664 10.1016/S1473-3099(19)30312-3 PMC8559573

[98] Chamilos G, Lewis RE, Kontoyiannis DP (2008) Delaying amphotericin B–based frontline therapy significantly increases mortality among patients with hematologic malignancy who have zygomycosis. Clin Infect Dis 47:503–509. 10.1086/590004 18611163 10.1086/590004

[99] Lanternier F, Poiree S, Elie C, Garcia-Hermoso D, Bakouboula P, Sitbon K et al (2015) Prospective pilot study of high-dose (10 mg/kg/day) liposomal amphotericin B (L-AMB) for the initial treatment of mucormycosis. J Antimicrob Chemother 70:3116–3123. 10.1093/jac/dkv236 26316385 10.1093/jac/dkv236

[100] Tashiro M, Namie H, Ito Y, Takazono T, Kakeya H, Miyazaki Y et al (2023) Prognostic association of liposomal amphotericin B doses above 5 mg/kg/d in mucormycosis: a nationwide epidemiologic and treatment analysis in Japan. Open Forum Infect Dis 10:ofad480. 10.1093/ofid/ofad480 37808895 10.1093/ofid/ofad480 PMC10552064

[101] van Burik J-AH, Hare RS, Solomon HF, Corrado ML, Kontoyiannis DP (2006) Posaconazole is effective as salvage therapy in zygomycosis: a retrospective summary of 91 cases. Clin Infect Dis 42:e61-65. 10.1086/500212 16511748 10.1086/500212

[102] Kyvernitakis A, Torres HA, Jiang Y, Chamilos G, Lewis RE, Kontoyiannis DP (2016) Initial use of combination treatment does not impact survival of 106 patients with haematologic malignancies and mucormycosis: a propensity score analysis. Clin Microbiol Infect 22:811.e1-811.e8. 10.1016/j.cmi.2016.03.029 27085727 10.1016/j.cmi.2016.03.029

[103] Spellberg B, Ibrahim A, Roilides E, Lewis RE, Lortholary O, Petrikkos G et al (2012) combination therapy for mucormycosis: why, what, and how? Clin Infect Dis 54:S73–S78. 10.1093/cid/cir885 22247449 10.1093/cid/cir885 PMC4542574

[104] Tedder M, Spratt JA, Anstadt MP, Hegde SS, Tedder SD, Lowe JE (1994) Pulmonary mucormycosis: results of medical and surgical therapy. Ann Thorac Surg 57:1044–1050. 10.1016/0003-4975(94)90243-7 8166512 10.1016/0003-4975(94)90243-7

[105] Kontoyiannis DP, Lewis RE (2011) How I treat mucormycosis. Blood 118:1216–1224. 10.1182/blood-2011-03-316430 21622653 10.1182/blood-2011-03-316430 PMC3292433

[106] Vaughan C, Bartolo A, Vallabh N, Leong SC (2018) A meta-analysis of survival factors in rhino-orbital-cerebral mucormycosis—has anything changed in the past 20 years? Clin Otolaryngol 43:1454–1464. 10.1111/coa.13175 29947167 10.1111/coa.13175

[107] Verma N, Singh S, Singh M, Chauhan A, Pradhan P, Jaiswal N et al (2022) Global epidemiological burden of fungal infections in cirrhosis patients: a systematic review with meta-analysis. Mycoses 65:266–284. 10.1111/myc.13387 34724269 10.1111/myc.13387

[108] Prattes J, Hoenigl M, Krause R, Buzina W, Valentin T, Reischies F et al (2017) Invasive aspergillosis in patients with underlying liver cirrhosis: a prospective cohort study. Med Mycol 55:803–812. 10.1093/mmy/myx011 28431001 10.1093/mmy/myx011

[109] Lahmer T, Peçanha-Pietrobom PM, Schmid RM, Colombo AL (2022) Invasive fungal infections in acute and chronic liver impairment: a systematic review. Mycoses 65:140–151. 10.1111/myc.13403 34837414 10.1111/myc.13403

[110] Fernández J, Acevedo J, Wiest R, Gustot T, Amoros A, Deulofeu C et al (2018) Bacterial and fungal infections in acute-on-chronic liver failure: prevalence, characteristics and impact on prognosis. Gut 67:1870–1880. 10.1136/gutjnl-2017-314240 28847867 10.1136/gutjnl-2017-314240

[111] Chen D, Qian Z, Su H, Meng Z, Lv J, Huang Y et al (2021) Invasive pulmonary aspergillosis in acute-on-chronic liver failure patients: short-term outcomes and antifungal options. Infect Dis Ther 10:2525–2538. 10.1007/s40121-021-00524-5 34468963 10.1007/s40121-021-00524-5 PMC8572893

[112] Levesque E, Ait-Ammar N, Dudau D, Clavieras N, Feray C, Foulet F et al (2019) Invasive pulmonary aspergillosis in cirrhotic patients: analysis of a 10-year clinical experience. Ann Intensive Care 9:31. 10.1186/s13613-019-0502-2 30778699 10.1186/s13613-019-0502-2 PMC6379500

[113] Pappas PG, Kauffman CA, Andes DR, Clancy CJ, Marr KA, Ostrosky-Zeichner L et al (2015) Clinical practice guideline for the management of candidiasis: 2016 update by the Infectious Diseases Society of America. Clin Infect Dis 62:e1-50. 10.1093/cid/civ933 26679628 10.1093/cid/civ933 PMC4725385

[114] Neofytos D, Fishman JA, Horn D, Anaissie E, Chang C-H, Olyaei A et al (2010) Epidemiology and outcome of invasive fungal infections in solid organ transplant recipients. Transpl Infect Dis 12:220–229. 10.1111/j.1399-3062.2010.00492.x 20113459 10.1111/j.1399-3062.2010.00492.x

[115] Neofytos D, Chatzis O, Nasioudis D, Boely Janke E, Doco Lecompte T, Garzoni C et al (2018) Epidemiology, risk factors and outcomes of invasive aspergillosis in solid organ transplant recipients in the Swiss Transplant Cohort Study. Transplant Infect Dis 20:20. 10.1111/tid.12898 10.1111/tid.12898 29668068

[116] Lockhart SR, Wagner D, Iqbal N, Pappas PG, Andes DR, Kauffman CA et al (2011) Comparison of in vitro susceptibility characteristics of *Candida* species from cases of invasive candidiasis in solid organ and stem cell transplant recipients: Transplant-Associated Infections Surveillance Network (TRANSNET), 2001 to 2006. J Clin Microbiol 49:2404–2410. 10.1128/JCM.02474-10 21562099 10.1128/JCM.02474-10 PMC3147873

[117] Fernández‐Ruiz M, Cardozo C, Salavert M, Aguilar‐Guisado M, Escolà‐Vergé L, Muñoz P, et al. (2019) Candidemia in solid organ transplant recipients in Spain: epidemiological trends and determinants of outcome. Transplant Infect Dis 21. 10.1111/tid.13195 10.1111/tid.13195 31610077

[118] Aguilar CA, Hamandi B, Fegbeutel C, Silveira FP, Verschuuren EA, Ussetti P et al (2018) Clinical risk factors for invasive aspergillosis in lung transplant recipients: results of an international cohort study. J Heart Lung Transplant 37:1226–1234. 10.1016/j.healun.2018.06.008 30139546 10.1016/j.healun.2018.06.008

[119] Shivasabesan G, Logan B, Brennan X, Lau C, Vaze A, Bennett M et al (2022) Disseminated *Aspergillus lentulus* infection in a heart transplant recipient: a case report. Clin Infect Dis 75:1235–1238. 10.1093/cid/ciac205 35275984 10.1093/cid/ciac205

[120] Pontes L, Gualtieri Beraquet CA, Arai T, Watanabe A, Moretti ML, Schreiber AZ (2022) Selection of Aspergillus fumigatus isolates carrying the G448S substitution in CYP51A gene after long-term treatment with voriconazole in an immunocompromised patient. Med Mycol Case Rep 36:5–9. 10.1016/j.mmcr.2022.02.002 35242508 10.1016/j.mmcr.2022.02.002 PMC8881195

[121] Gavalda J, Len O, San Juan R, Aguado JM, Fortun J, Lumbreras C et al (2005) Risk factors for invasive aspergillosis in solid-organ transplant recipients: a case-control study. Clin Infect Dis 41:52–59. 10.1086/430602 15937763 10.1086/430602

[122] Gavaldà J, Meije Y, Fortún J, Roilides E, Saliba F, Lortholary O et al (2014) Invasive fungal infections in solid organ transplant recipients. Clin Microbiol Infect 20:27–48. 10.1111/1469-0691.12660 24810152 10.1111/1469-0691.12660

[123] Pennington KM, Yost KJ, Escalante P, Razonable RR, Kennedy CC (2019) Antifungal prophylaxis in lung transplant: a survey of United States’ transplant centers. Clin Transplant 33:33. 10.1111/ctr.13630 10.1111/ctr.13630 PMC6635088 31173402

[124] Cruciani M, Mengoli C, Malena M, Bosco O, Serpelloni G, Grossi P (2006) Antifungal prophylaxis in liver transplant patients: a systematic review and meta-analysis. Liver Transpl 12:850–858. 10.1002/lt.20690 16628697 10.1002/lt.20690

[125] Eschenauer GA, Kwak EJ, Humar A, Potoski BA, Clarke LG, Shields RK et al (2015) Targeted versus universal antifungal prophylaxis among liver transplant recipients. Am J Transplant 15:180–189. 10.1111/ajt.12993 25359455 10.1111/ajt.12993 PMC4365781

[126] Giannella M, Husain S, Saliba F, Viale P (2018) Use of echinocandin prophylaxis in solid organ transplantation. J Antimicrob Chemother 73:i51–i59. 10.1093/jac/dkx449 29304212 10.1093/jac/dkx449

[127] Winston DJ, Limaye AP, Pelletier S, Safdar N, Morris MI, Meneses K et al (2014) Randomized, double-blind trial of anidulafungin versus fluconazole for prophylaxis of invasive fungal infections in high-risk liver transplant recipients. Am J Transplant 14:2758–2764. 10.1111/ajt.12963 25376267 10.1111/ajt.12963

[128] Evans JDW, Morris PJ, Knight SR (2014) antifungal prophylaxis in liver transplantation: a systematic review and network meta-analysis. Am J Transplant 14:2765–2776. 10.1111/ajt.12925 25395336 10.1111/ajt.12925

[129] Fortún J, Muriel A, Martín-Dávila P, Montejo M, Len O, Torre-Cisneros J et al (2016) Caspofungin versus fluconazole as prophylaxis of invasive fungal infection in high-risk liver transplantation recipients: a propensity score analysis. Liver Transpl 22:427–435. 10.1002/lt.24391 26709146 10.1002/lt.24391

[130] Rinaldi M, Bartoletti M, Ferrarese A, Franceschini E, Campoli C, Coladonato S et al (2021) Breakthrough invasive fungal infection after liver transplantation in patients on targeted antifungal prophylaxis: A prospective multicentre study. Transplant Infectious Disease 23:23. 10.1111/tid.13608 10.1111/tid.13608 PMC8519035 33768656

[131] Gatti M, Rinaldi M, Ferraro G, Toschi A, Caroccia N, Arbizzani F et al (2021) Breakthrough invasive fungal infections in liver transplant recipients exposed to prophylaxis with echinocandins vs other antifungal agents: a systematic review and meta-analysis. Mycoses 64:1317–1327. 10.1111/myc.13362 34387004 10.1111/myc.13362 PMC9292189

[132] Giannella M, Ercolani G, Cristini F, Morelli M, Bartoletti M, Bertuzzo V et al (2015) High-dose weekly liposomal amphotericin B antifungal prophylaxis in patients undergoing liver transplantation. Transplantation 99:848–854. 10.1097/TP.0000000000000393 25531982 10.1097/TP.0000000000000393

[133] Thompson GR, Young J-AH (2021) Aspergillus infections. N Engl J Med 385:1496–1509. 10.1056/NEJMra2027424 34644473 10.1056/NEJMra2027424

[134] Fernández-Ruiz M, Bodro M, Gutiérrez Martín I, Rodriguez-Álvarez R, Ruiz-Ruigómez M, Sabé N et al (2023) Isavuconazole for the treatment of invasive mold disease in solid organ transplant recipients: a multicenter study on efficacy and safety in real-life clinical practice. Transplantation 107:762–773. 10.1097/TP.0000000000004312 36367924 10.1097/TP.0000000000004312

[135] Kozuch JM, Burt C, Afshar K, Aslam S, Yung G, Mariski M et al (2024) Difference in immunosuppressant dose requirement when transitioning to isavuconazole from other azoles in thoracic transplant recipients. Transplant Infect Dis 26:26. 10.1111/tid.14209 10.1111/tid.14209 38059638

[136] Cojutti PG, Rinaldi M, Giannella M, Viale P, Pea F (2023) Successful and safe real-time TDM-guided treatment of invasive pulmonary and cerebral aspergillosis using low-dose isavuconazole in a patient with primary biliary cirrhosis: grand round/a case study. Ther Drug Monit 45:140–142. 10.1097/FTD.0000000000001064 36728593 10.1097/FTD.0000000000001064

[137] Paiva J-A, Pereira JM (2023) Treatment of invasive candidiasis in the era of Candida resistance. Curr Opin Crit Care 29:457–462. 10.1097/MCC.0000000000001077 37641511 10.1097/MCC.0000000000001077

[138] Little JS, Kampouri E, Friedman DZ, McCarty T, Thompson GR, Kontoyiannis DP et al (2024) The burden of invasive fungal disease following chimeric antigen receptor T-cell therapy and strategies for prevention. Open Forum Infect Dis 11:ofae133. 10.1093/ofid/ofae133 38887472 10.1093/ofid/ofae133 PMC11181190

[139] Stemler J, Mellinghoff SC, Khodamoradi Y, Sprute R, Classen AY, Zapke SE et al (2023) Primary prophylaxis of invasive fungal diseases in patients with haematological malignancies: 2022 update of the recommendations of the Infectious Diseases Working Party (AGIHO) of the German Society for Haematology and Medical Oncology (DGHO). J Antimicrob Chemother 78:1813–1826. 10.1093/jac/dkad143 37311136 10.1093/jac/dkad143 PMC10393896

[140] Ullmann AJ, Lipton JH, Vesole DH, Chandrasekar P, Langston A, Tarantolo SR et al (2007) Posaconazole or fluconazole for prophylaxis in severe graft-versus-host disease. N Engl J Med 356:335–347. 10.1056/NEJMoa061098 17251530 10.1056/NEJMoa061098

[141] Cornely OA, Maertens J, Winston DJ, Perfect J, Ullmann AJ, Walsh TJ et al (2007) Posaconazole vs. fluconazole or itraconazole prophylaxis in patients with neutropenia. N Engl J Med 356:348–359. 10.1056/NEJMoa061094 17251531 10.1056/NEJMoa061094

[142] Maertens JA, Girmenia C, Brüggemann RJ, Duarte RF, Kibbler CC, Ljungman P, et al. (2018) European guidelines for primary antifungal prophylaxis in adult haematology patients: summary of the updated recommendations from the European Conference on Infections in Leukaemia. J Antimicrob Chemother 10.1093/jac/dky286 10.1093/jac/dky286 30085172

[143] Cornely OA, Hoenigl M, Lass-Flörl C, Chen SC-A, Kontoyiannis DP, Morrissey CO et al (2019) Defining breakthrough invasive fungal infection–position paper of the mycoses study group education and research consortium and the European Confederation of Medical Mycology. Mycoses 62:716–729. 10.1111/myc.12960 31254420 10.1111/myc.12960 PMC6692208

[144] Hong JY, Kang C-I, Yang J, Ko J-H, Huh K, Cho SY et al (2023) Breakthrough invasive fungal infection in patients with myeloid malignancy receiving posaconazole tablet prophylaxis: clinical features, risk factors, and posaconazole profiles. Med Mycol 61:61. 10.1093/mmy/myad046 10.1093/mmy/myad046 37120735

[145] Auberger J, Lass-Florl C, Aigner M, Clausen J, Gastl G, Nachbaur D (2012) Invasive fungal breakthrough infections, fungal colonization and emergence of resistant strains in high-risk patients receiving antifungal prophylaxis with posaconazole: real-life data from a single-centre institutional retrospective observational study. J Antimicrob Chemother 67:2268–2273. 10.1093/jac/dks189 22653819 10.1093/jac/dks189

[146] Kontoyiannis DP, Lionakis MS, Lewis RE, Chamilos G, Healy M, Perego C et al (2005) Zygomycosis in a tertiary-care cancer center in the Era of *Aspergillus-* active antifungal therapy: a case-control observational study of 27 recent cases. J Infect Dis 191:1350–1360. 10.1086/428780 15776383 10.1086/428780

[147] Ananda-Rajah MR, Grigg A, Downey MT, Bajel A, Spelman T, Cheng A et al (2012) Comparative clinical effectiveness of prophylactic voriconazole/posaconazole to fluconazole/itraconazole in patients with acute myeloid leukemia/myelodysplastic syndrome undergoing cytotoxic chemotherapy over a 12-year period. Haematologica 97:459–463. 10.3324/haematol.2011.051995 22058198 10.3324/haematol.2011.051995 PMC3291603

[148] Tormo M, Pérez-Martínez A, Calabuig M, Hernández-Boluda JC, Amat P, Navarro D et al (2018) Primary prophylaxis of invasive fungal infections with posaconazole or itraconazole in patients with acute myeloid leukaemia or high-risk myelodysplastic syndromes undergoing intensive cytotoxic chemotherapy: a real-world comparison. Mycoses 61:206–212. 10.1111/myc.12728 29125660 10.1111/myc.12728

[149] Cho S, Lee D, Choi S, Choi J, Lee H, Kim S et al (2015) Posaconazole for primary antifungal prophylaxis in patients with acute myeloid leukaemia or myelodysplastic syndrome during remission induction chemotherapy: a single-centre retrospective study in Korea and clinical considerations. Mycoses 58:565–571. 10.1111/myc.12357 26214656 10.1111/myc.12357

[150] Hachem R, Assaf A, Numan Y, Shah P, Jiang Y, Chaftari A-M et al (2017) Comparing the safety and efficacy of voriconazole versus posaconazole in the prevention of invasive fungal infections in high-risk patients with hematological malignancies. Int J Antimicrob Agents 50:384–388. 10.1016/j.ijantimicag.2017.03.021 28694233 10.1016/j.ijantimicag.2017.03.021

[151] Park WB, Kim N-H, Kim K-H, Lee SH, Nam W-S, Yoon SH et al (2012) The effect of therapeutic drug monitoring on safety and efficacy of voriconazole in invasive fungal infections: a randomized controlled trial. Clin Infect Dis 55:1080–1087. 10.1093/cid/cis599 22761409 10.1093/cid/cis599

[152] Lindsay J, Krantz EM, Morris J, Sweet A, Tverdek F, Joshi A et al (2022) Voriconazole in hematopoietic stem cell transplantation and cellular therapies: real-world usage and therapeutic level attainment at a major transplantation center. Transplant Cell Ther 28:511.e1-511.e10. 10.1016/j.jtct.2022.05.030 35623614 10.1016/j.jtct.2022.05.030

[153] Höhl R, Bertram R, Kinzig M, Haarmeyer G, Baumgärtel M, Geise A et al (2022) Isavuconazole therapeutic drug monitoring in critically ill ICU patients: a monocentric retrospective analysis. Mycoses 65:747–752. 10.1111/myc.13469 35535740 10.1111/myc.13469

[154] Bertram R, Naumann H, Bartsch V, Hitzl W, Kinzig M, Haarmeyer G et al (2023) Clinical and demographic factors affecting trough levels of isavuconazole in critically ill patients with or without COVID-19. Mycoses 66:1071–1078. 10.1111/myc.13653 37700457 10.1111/myc.13653

[155] Mikulska M, Melchio M, Signori A, Ullah N, Miletich F, Sepulcri C et al (2024) Lower blood levels of isavuconazole in critically ill patients compared with other populations: possible need for therapeutic drug monitoring. J Antimicrob Chemother. 10.1093/jac/dkae037 38366368 10.1093/jac/dkae037

[156] McCreary EK, Davis MR, Narayanan N, Andes DR, Cattaneo D, Christian R et al (2023) Utility of triazole antifungal therapeutic drug monitoring: insights from the Society of Infectious Diseases Pharmacists. Pharmacother: J Hum Pharmacol Drug Ther 43:1043–1050. 10.1002/phar.2850 10.1002/phar.2850 37459118

[157] Maertens J, Lodewyck T, Donnelly JP, Chantepie S, Robin C, Blijlevens N et al (2023) Empiric vs preemptive antifungal strategy in high-risk neutropenic patients on fluconazole prophylaxis: a randomized trial of the European Organization for Research and Treatment of Cancer. Clin Infect Dis 76:674–682. 10.1093/cid/ciac623 35906831 10.1093/cid/ciac623 PMC9938744

[158] Vergidis P, Clancy CJ, Shields RK, Park SY, Wildfeuer BN, Simmons RL et al (2016) Intra-abdominal Candidiasis: the importance of early source control and antifungal treatment. PLoS ONE 11:e0153247. 10.1371/journal.pone.0153247 27123857 10.1371/journal.pone.0153247 PMC4849645

[159] Maseda E, Martín-Loeches I, Zaragoza R, Pemán J, Fortún J, Grau S et al (2023) Critical appraisal beyond clinical guidelines for intraabdominal candidiasis. Crit Care 27:382. 10.1186/s13054-023-04673-6 37789338 10.1186/s13054-023-04673-6 PMC10546659

[160] Martin-Loeches I, Antonelli M, Cuenca-Estrella M, Dimopoulos G, Einav S, De Waele JJ et al (2019) ESICM/ESCMID task force on practical management of invasive candidiasis in critically ill patients. Intensive Care Med. 10.1007/s00134-019-05599-w 30911804 10.1007/s00134-019-05599-w

[161] Falcone M, Tiseo G, Gutiérrez-Gutiérrez B, Raponi G, Carfagna P, Rosin C et al (2019) Impact of initial antifungal therapy on the outcome of patients with candidemia and septic shock admitted to medical wards: a propensity score–adjusted analysis. Open Forum Infect Dis 6:ofz251. 10.1093/ofid/ofz251 31334296 10.1093/ofid/ofz251 PMC6634434

[162] Pittet D, Monod M, Suter PM, Frenk E, Auckenthaler R (1994) Candida colonization and subsequent infections in critically iii surgical patients. Ann Surg 220:751–758. 10.1097/00000658-199412000-00008 7986142 10.1097/00000658-199412000-00008 PMC1234477

[163] Keane S, Geoghegan P, Povoa P, Nseir S, Rodriguez A, Martin-Loeches I (2018) Systematic review on the first line treatment of amphotericin B in critically ill adults with candidemia or invasive candidiasis. Expert Rev Anti Infect Ther 16:839–847. 10.1080/14787210.2018.1528872 30257597 10.1080/14787210.2018.1528872

[164] Gioia F, Gomez-Lopez A, Alvarez ME, Gomez-García de la Pedrosa E, Martín-Davila P, Cuenca-Estrella M et al (2020) Pharmacokinetics of echinocandins in suspected candida peritonitis: a potential risk for resistance. Int J Infect Dis 101:24–28. 10.1016/j.ijid.2020.09.019 32937195 10.1016/j.ijid.2020.09.019

[165] Welte R, Oberacher H, Gasperetti T, Pfisterer H, Griesmacher A, Santner T et al (2021) Pharmacokinetics and antifungal activity of echinocandins in ascites fluid of critically ill patients. Antimicrob Agents Chemother 65:65. 10.1128/AAC.02565-20 10.1128/AAC.02565-20 PMC8218616 33972242

[166] Liu X, Liu D, Pan Y, Li Y (2020) Pharmacokinetic/pharmacodynamics variability of echinocandins in critically ill patients: a systematic review and meta-analysis. J Clin Pharm Ther 45:1207–1217. 10.1111/jcpt.13211 32672361 10.1111/jcpt.13211 PMC7689702

[167] Garbez N, Mbatchi LC, Wallis SC, Muller L, Lipman J, Roberts JA et al (2022) Caspofungin population pharmacokinetic analysis in plasma and peritoneal fluid in septic patients with intra-abdominal infections: a prospective cohort study. Clin Pharmacokinet 61:673–686. 10.1007/s40262-021-01062-6 34931282 10.1007/s40262-021-01062-6

[168] Garbez N, Mbatchi L, Wallis SC, Muller L, Lipman J, Roberts JA et al (2021) Prospective cohort study of micafungin population pharmacokinetic analysis in plasma and peritoneal fluid in septic patients with intra-abdominal infections. Antimicrob Agents Chemother 65:65. 10.1128/AAC.02307-20 10.1128/AAC.02307-20 PMC8218641 33846133

[169] Martial LC, ter Heine R, Schouten JA, Hunfeld NG, van Leeuwen HJ, Verweij PE et al (2017) Population pharmacokinetic model and pharmacokinetic target attainment of micafungin in intensive care unit patients. Clin Pharmacokinet 56:1197–1206. 10.1007/s40262-017-0509-5 28144840 10.1007/s40262-017-0509-5 PMC5591795

[170] Wasylyshyn A, Stoneman EK (2024) Management of *Candida auris*. JAMA 331:611. 10.1001/jama.2023.24921 38252429 10.1001/jama.2023.24921

[171] Jaggavarapu S, Burd EM, Weiss DS (2020) Micafungin and amphotericin B synergy against Candida auris. Lancet Microbe 1:e314–e315. 10.1016/S2666-5247(20)30194-4 35544183 10.1016/S2666-5247(20)30194-4

[172] Yan T, Li S, Ou H, Zhu S, Huang L, Wang D (2020) appropriate source control and antifungal therapy are associated with improved survival in critically ill surgical patients with intra-abdominal Candidiasis. World J Surg 44:1459–1469. 10.1007/s00268-020-05380-x 31965275 10.1007/s00268-020-05380-x

